# One-pot solution plasma synthesis of tungsten carbide core–shell nanoparticles for efficient conversion of cellulose to lactic acid

**DOI:** 10.1039/d5ra09791f

**Published:** 2026-07-02

**Authors:** Kouki Yamamoto, Yuki Atsuumi, Akinari Uesugi, Taketo Imamura, Toshiki Iwato, Gasidit Panomsuwan, Takahiro Ishizaki

**Affiliations:** a Materials Science and Engineering, Graduate School of Engineering and Science, Shibaura Institute of Technology Toyosu, Koto-Ku Tokyo 135-8548 Japan; b Department of Materials Engineering, Faculty of Engineering, Kasetsart University Bangkok 10900 Thailand; c College of Engineering, Shibaura Institute of Technology Toyosu, Koto-Ku Tokyo 135-8548 Japan ishizaki@shibaura-it.ac.jp

## Abstract

The valorization of lignocellulosic biomass into high-value chemicals has garnered significant attention as a sustainable alternative to fossil-fuel-based processes. In particular, the catalytic conversion of cellulose into lactic acid is of great importance, as it serves as a precursor for biodegradable polylactic acid (PLA). While tungsten (W)-based catalysts are known to promote the retro-aldol reaction necessary for lactic acid production, conventional synthesis of carbide catalysts often requires energy-intensive high-temperature processes. In this study, we demonstrated a facile, one-pot synthesis of tungsten carbide (WC_1−*x*_) nanoparticles highly dispersed on a carbon matrix using a solution plasma (SP) process. By regulating the pulse frequency during the SP treatment, the W content and dispersion in the carbon support were successfully controlled. Structural analyses revealed that the WC_1−*x*_ nanoparticles were encapsulated within carbon shells, preventing oxidation and aggregation. The synthesized WC_1−*x*_/C catalyst exhibited superior catalytic performance for the hydrothermal conversion of cellulose compared to commercial tungsten carbides. Under optimized conditions (190 °C, 48 h), a lactic acid yield of approximately 14.0% was achieved. To assess the catalytic stability, reusability tests and post-reaction characterizations were conducted. While ICP-OES analysis after the first cycle showed negligible W leaching, a significant decrease in yield was observed over four consecutive cycles. XRD and SEM analyses revealed that this deactivation was primarily due to the loss of crystallinity and structural degradation of the WC_1−*x*_ phase under harsh hydrothermal conditions. This work provides a novel and rapid route for developing efficient non-noble metal catalysts and clarifies the factors influencing their stability during biomass valorization.

## Abstract 2

WC_1−*x*_/carbon core–shell nanoparticles were synthesized *via* a one-pot solution plasma process in benzene. These catalysts facilitate the hydrothermal conversion of cellulose into lactic acid with a maximum yield of 14.0%, offering a green route for biomass valorization.

## Graphical abstract

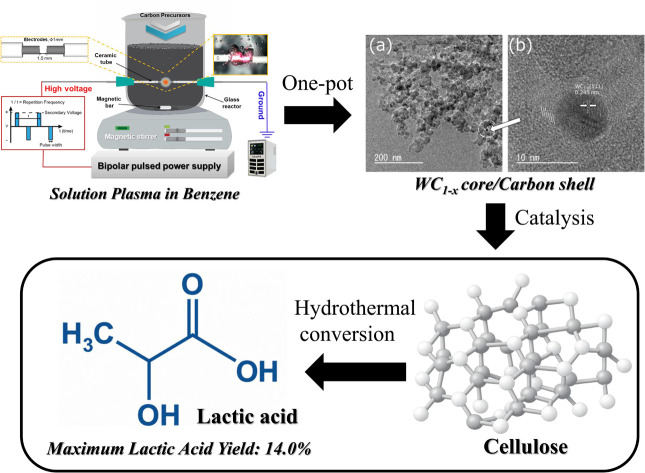

## 1. Introduction

The escalating emission of greenhouse gases (GHGs), such as carbon dioxide (CO_2_), methane, and nitrous oxide, stemming from fossil fuel consumption, presents a critical global environmental challenge.^1^ Consequently, the effective utilization of biomass as a sustainable alternative to fossil fuels is urgently demanded. Among biomass resources, lignocellulosic biomass, such as wood, attracts particular attention as a carbon-neutral and abundant resource that absorbs CO_2_ during growth and supports sustainability through afforestation.^2–6^ In recent years, significant research efforts have focused on converting cellulose, the primary component of wood, into high-value-added chemicals. In particular, lactic acid, a precursor for the biodegradable plastic polylactic acid (PLA), is regarded as a vital target compound from the perspective of green chemistry.^7–13^

Currently, the industrial production of lactic acid relies primarily on the fermentation of carbohydrates.^14,15^ Although fermentation is an established process yielding high purity and yield, it often utilizes edible biomass like corn, raising concerns regarding competition with the food supply.^15^ Furthermore, this method faces environmental and economic challenges, including long fermentation times, the need for strict bacterial management, and the generation of large quantities of gypsum (calcium sulfate) waste during the pH adjustment step.^8,14^ As an alternative to overcome these drawbacks, the chemocatalytic hydrothermal conversion of cellulose using solid catalysts has garnered significant attention.^16^ This approach offers a potentially cheaper and more environmentally benign process, enabling the rapid, one-step synthesis of lactic acid from non-edible cellulose while minimizing waste generation. However, current chemocatalytic methods suffer from lower yields and selectivity compared to fermentation, necessitating the development of high-performance catalysts.

In cellulose hydrolysis, the contact efficiency between the solid catalyst and the solid substrate (cellulose) significantly influences the reaction rate. Carbon materials are reported to exhibit excellent affinity for cellulose due to their surface functional groups and high wettability, which facilitate hydrogen bonding with the hydroxyl groups of cellulose^17^ and enhance solid–solid interfacial contact. Meanwhile, tungsten (W)-based compounds possessing Lewis acid sites are known to be effective for converting glucose and other intermediates into lactic acid (*e.g.*, *via* the retro-aldol reaction).^16^ Indeed, W-containing catalysts such as AlW and ZrW have been reported to function effectively.^16^ Therefore, a composite material comprising active tungsten species highly dispersed on a carbon support with superior cellulose adsorption capability is considered an ideal catalyst system for this reaction. Among tungsten compounds, tungsten carbides (*e.g.*, WC, W_2_C) are promising candidates due to their high acid resistance and catalytic properties similar to noble metals.^18^ However, conventional synthesis of tungsten carbides typically requires high temperatures, often exceeding 1000 °C, or complex carburization processes under flammable atmospheres, leading to high energy consumption.^19,20^ Although recent progress has been made in lowering synthesis temperatures, there is still a need for more energy-efficient and simpler synthesis routes. For instance, traditional carburization often requires temperatures exceeding 1000 °C and prolonged heating times, which significantly hinders industrial implementation from an energy-efficiency standpoint.^21^ In contrast, the development of a low-temperature synthesis route is highly desirable. Thus, there is a demand for novel processes that can synthesize these materials simply under milder conditions.

Based on this background, we focused on the solution plasma (SP) process, a liquid-phase plasma method. The SP process generates non-equilibrium plasma by applying high-voltage pulses between electrodes submerged in a solution. In this high-energy reaction field, active species such as radicals generated from the solvent and electrode materials react instantaneously, enabling the rapid synthesis of nanomaterials like carbon and metal carbides in liquid at ambient temperature and pressure.^22–24^ Specifically, by generating SP using tungsten electrodes in an organic solvent, carbon generation from solvent decomposition and W sputtering from the electrodes occur simultaneously. This allows for the “one-pot” synthesis of carbon composites with dispersed W-based nanoparticles.^25,26^ This method offers the advantage of simple and rapid catalyst preparation without the need for high-temperature heating or reducing gases required by conventional methods. Thus, our SP-based approach enables the synthesis of WC catalysts in a liquid phase at ambient temperature within minutes. This significant reduction in energy input and processing time highlights the novelty and potential of the proposed method for sustainable catalyst development.

In this study, we aimed to synthesize tungsten carbide nanoparticle-dispersed carbon catalysts (WC/C) using the SP process in an organic solvent. The effects of pulse frequency control on the W content and particle characteristics were investigated. Furthermore, the catalytic effectiveness of the synthesized WC/C materials for lactic acid production was evaluated through the hydrothermal conversion of cellulose.

## 2. Experimental

### 2.1 Synthesis of tungsten compound-dispersed carbon

**Fig. 1:**
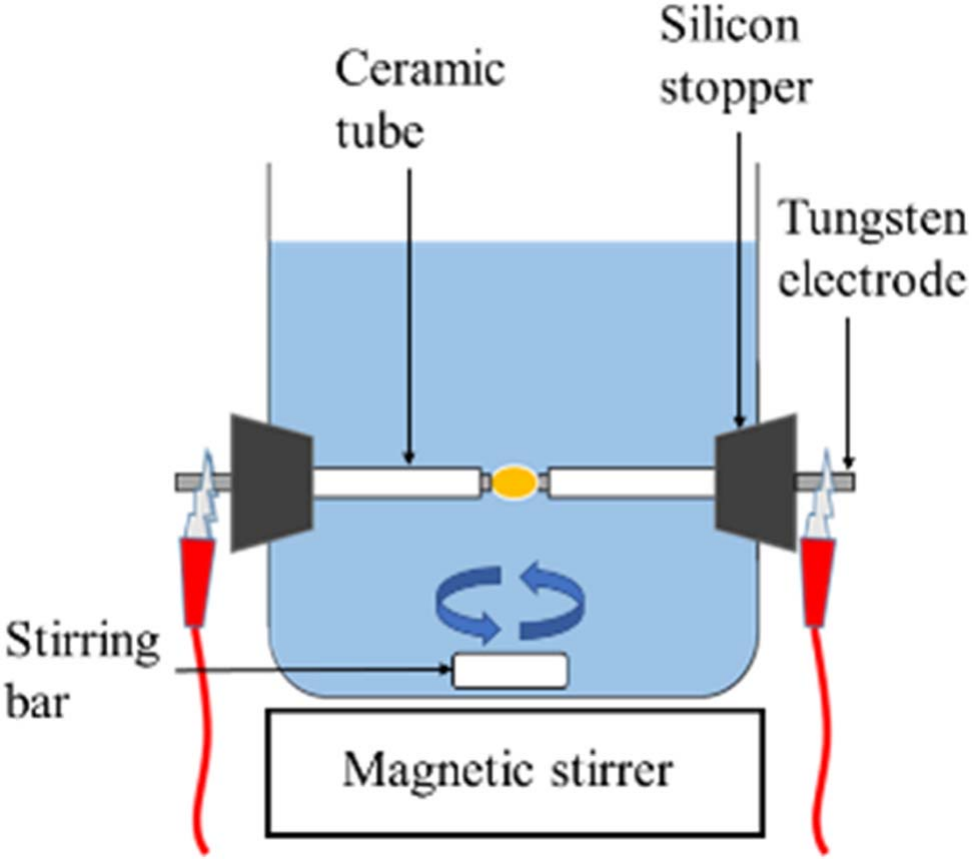
Schematic diagram of solution plasma equipment.

Tungsten compound-dispersed carbon materials were synthesized *via* a solution plasma (SP) process using tungsten electrodes in benzene. A tungsten rod (diameter: 1 mm, purity >99.95%, Nilaco Corp.) was used as the electrode, and 100 mL of benzene (Kanto Chemical Co., Inc.) was used as the solvent. A schematic diagram of the SP apparatus is shown in Fig. 1. The SP treatment was conducted with a pulse width of 1 µs and an electrode gap of 1 mm. The discharge was maintained for 20 min at four different frequencies: 12.5, 25.0, 37.5, and 50.0 kHz. After the reaction, the solution was suction-filtered using a membrane filter (pore size: 0.2 µm, Merck) and washed with acetone. The obtained solid was dried in an electric furnace at 100 °C for 24 h and then ground into a powder using an agate mortar. Hereafter, the samples synthesized at frequencies of 12.5, 25.0, 37.5, and 50.0 kHz are denoted as Benzen_W_12.5, Benzen_W_25.0, Benzen_W_37.5, and Benzen_W_50.0, respectively. The detailed SP synthesis conditions are summarized in Table 1. For comparison, a tungsten-free carbon sample was synthesized using carbon fiber electrodes (diameter: 1 mm) under the same conditions at a frequency of 12.5 kHz. This control sample is denoted as Benzen_C_12.5.

**Table 1:** Solution plasma conditions

Solvent	Benzene				
Electrode	W				
Applied voltage [kV]	1.5				
Frequency [kHz]	12.5	12.5	25.0	37.5	50.0
Pulse width [µs]	0.5				
Distance between electrodes [mm]	1.0				
Processing time [min]	20				
Stirring rotation speed [rpm]	500				

### 2.2 Characterization

The surface morphology and elemental composition of the synthesized samples were examined using a field-emission scanning electron microscope (FE-SEM; JSM-7610F, JEOL) equipped with an energy-dispersive X-ray spectrometer (EDS). The microstructure was analyzed using transmission electron microscopy (TEM; JEM-2100/HR, JEOL). The tungsten content in the samples was calculated based on the mass balance, determined by measuring the weight change of the electrodes before and after the SP process and the total weight of the recovered sample using a precision electronic balance (Cubis series, Sartorius). The crystal structures were identified by X-ray diffraction (XRD; SmartLab, Rigaku) using Cu Kα radiation. The measurements were performed in a 2*θ* range of 5–90° with a scan speed of 10° min^−1^. Raman spectra were obtained by a NRS-5100 spectrometer (JASCO) using an excitation wavelength of 532.1 nm. The chemical bonding states were evaluated using X-ray photoelectron spectroscopy (XPS; JPS-9010 MC, JEOL) and Fourier transform infrared spectroscopy (FT-IR; IRTracer-100, Shimadzu). XPS measurements were carried out using Mg Kα radiation (10 kV, 25 mA), and the binding energies were calibrated referencing the C 1s peak at 284.5 eV. The mass fractions of carbon, nitrogen, and hydrogen in the carbon powder were determined using an organic elemental analyzer (CHNS/O Analyzer 2400 II series, PerkinElmer Japan Co., Ltd). The specific surface area and pore size distribution were calculated from N_2_ adsorption–desorption isotherms at 77 K using a surface area analyzer (TriStar II 3020, Micromeritics). Before the measurements, the samples were degassed at 100 °C for 24 h.

### 2.3 Catalytic performance evaluation

**Fig. 2:**
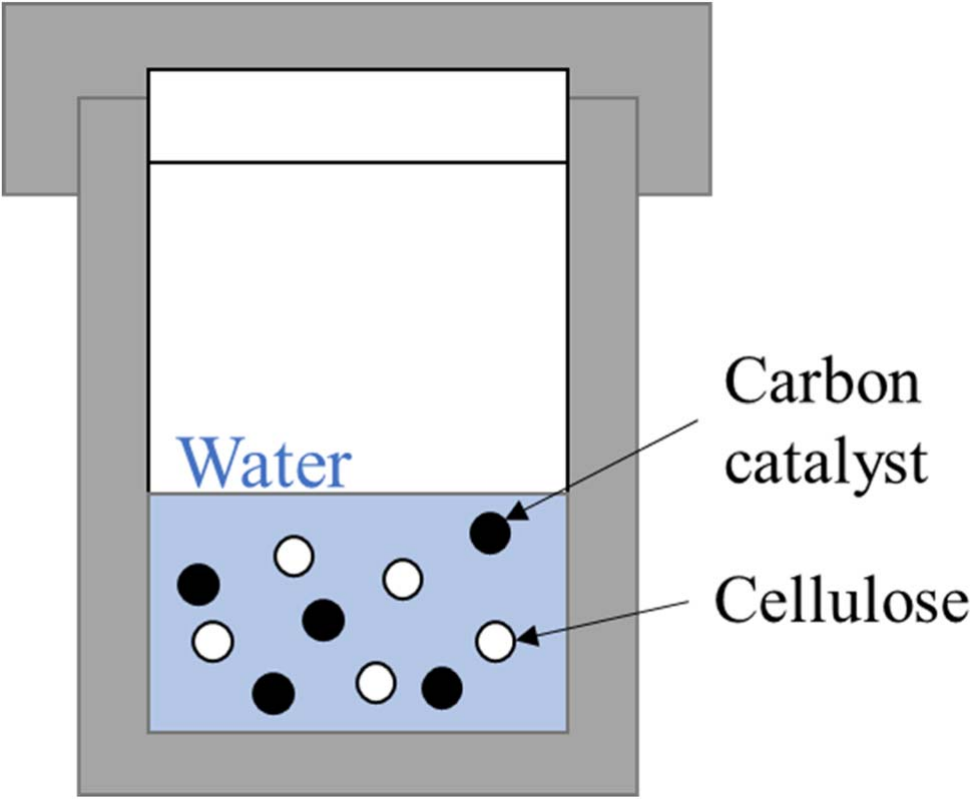
Schematic diagram of thermal hydrolysis test for cellulose.

The catalytic performance of the synthesized samples was evaluated *via* the hydrothermal conversion of cellulose. The reaction conditions and a schematic illustration of the process are shown in Table 2 and Fig. 2, respectively. Cellulose powder (particle size <38 µm, Fujifilm Wako Pure Chemical Corp.) was used as the substrate and pretreated by ball milling for 48 h. The catalyst, pretreated cellulose, and ultrapure water were placed in a 15 mL autoclave. After thorough stirring, the mixture was heated in a convection electric furnace at a specified temperature. After the reaction, the suspension was filtered through a membrane filter (pore size: 0.1 µm, Merck) to remove the solid residues (catalyst and unreacted cellulose), and the filtrate was collected. The quantitative analysis of lactic acid and the identification of by-products were performed using high-performance liquid chromatography (HPLC; CBM-20A system, Shimadzu) equipped with a Rezex ROA-Organic Acid column (Phenomenex). The mobile phase was 0.005 N H_2_SO_4_. The lactic acid concentration was determined using a calibration curve prepared with standard solutions. The lactic acid yield was calculated based on the carbon mass balance between the initial cellulose and the produced lactic acid. The carbon content of the cellulose was determined to be 42 wt% by CHNS analysis using an elemental analyzer (2400 Series II, PerkinElmer). The yield was calculated using the following equation:1
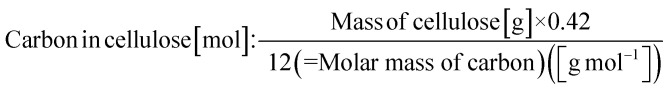
2
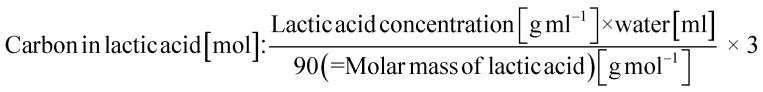
3
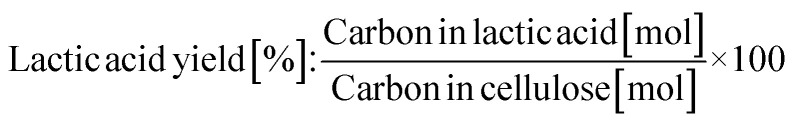


**Table 2:** Thermal hydrolysis conditions of cellulose

Catalyst [mg]	50			
Cellulose [mg]	125			
Water [ml]	5			
Heat treatment time [h]	12	24	36	48
Temperature [°C]	190			

## 3. Results and discussion

### 3.1 Structural and morphological characterization

The surface morphologies of the samples synthesized at different frequencies (12.5, 25.0, 37.5, and 50.0 kHz) were examined using FE-SEM (Fig. S1). Regardless of the frequency applied, primary particles with diameters of tens of nanometers were observed to aggregate, forming secondary particles of several hundred nanometers in all samples. Elemental mapping images (Fig. S2) revealed a uniform dispersion of C, W, and O throughout the samples.

**Fig. 3:**
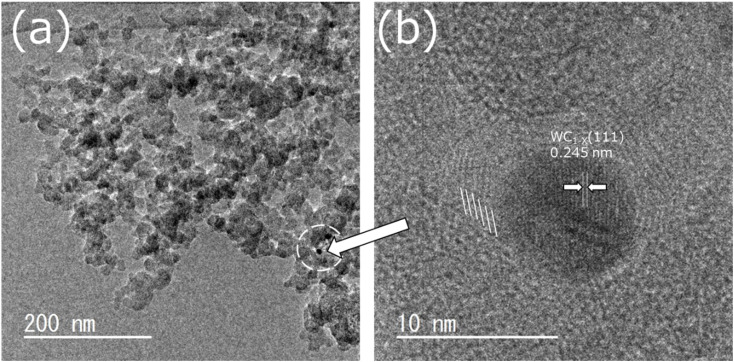
(a) TEM image of a sample synthesized at 12.5 kHz and (b) enlarged TEM image at open circle point in Fig. 3(a).

The presence of oxygen is consistent with the FT-IR results discussed later, suggesting the introduction of oxygen-containing functional groups onto the carbon support surface. To elucidate the microstructure of the catalyst, TEM observation was performed on a representative sample (Benzene_W_12.5, Fig. 3). Numerous spherical dark-contrast particles with a diameter of approximately 7 nm were observed dispersed within particles of tens of nanometers. The lattice spacing in the high-resolution images matched the (111) plane of cubic WC_1−*x*_, identifying these nanoparticles as tungsten carbide. Notably, a core–shell structure was confirmed, in which the WC_1−*x*_ nanoparticles were encapsulated by approximately six layers of graphene (carbon shell). The tungsten carbide nanoparticles were observed to be dispersed on the carbon matrix. The size distribution of these nanoparticles was quantified by measuring approximately 100 particles from the TEM images. As shown in the histogram in Fig. S3, the nanoparticles exhibit a narrow size distribution with an average diameter of 7.73 ± 0.19 nm, confirming the high dispersion and size uniformity achieved by the solution plasma process. These results clearly demonstrate that the present solution plasma process successfully formed a composite material in which fine WC_1−*x*_ nanoparticles are highly dispersed within a carbon matrix.

### 3.2 Composition and crystal structure analysis

**Fig. 4:**
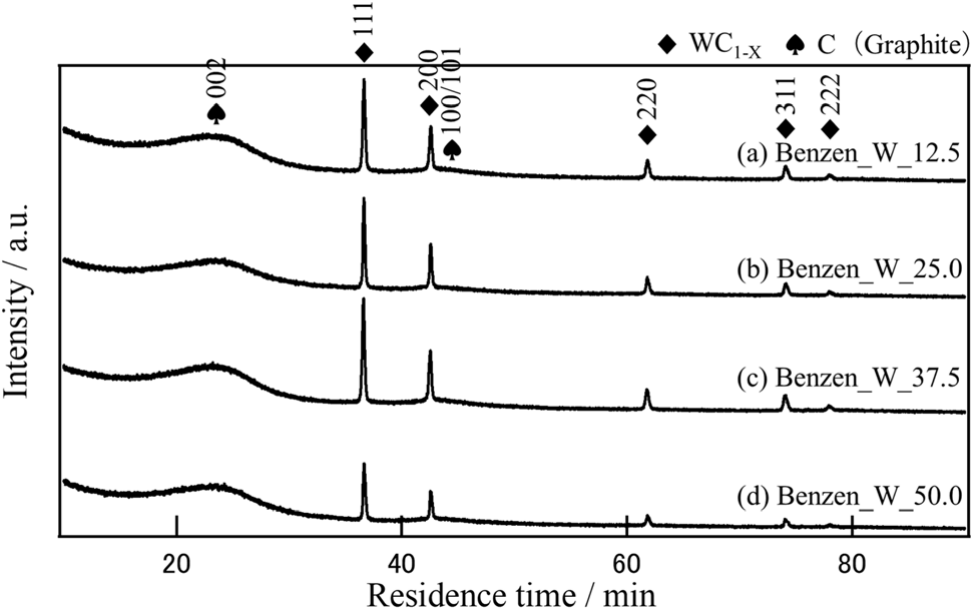
XRD patterns of samples synthesized at (a) 12.5, (b) 25.0, (c) 37.5, and (d) 50.0 kHz.

Table 3 presents the tungsten content of each sample. A trend was observed where the W content increased as the frequency decreased, with the sample synthesized at 12.5 kHz exhibiting the highest value (4.03 wt%). This is presumably because, at higher frequencies, the rate of carbon generation from benzene decomposition relatively outweighed the sputtering rate of the tungsten electrode (W supply). The elemental composition of the synthesized carbon samples was quantified using CHNS/O analysis, as summarized in Table S1. Carbon, hydrogen, and oxygen were detected in all samples, indicating that the constituent elements of the precursors were successfully incorporated into the carbon framework. The presence of O and H is primarily attributed to the formation of surface functional groups, such as hydroxyl (–OH) groups, on the carbon surface. This interpretation is consistent with the subsequent characterization results (FT-IR), which suggest that these elements originate from the residual functionalization of the precursor or environmental adsorption. Fig. 4 shows the XRD patterns of the synthesized samples. In all samples, a broad diffraction peak derived from the graphite (002) plane was observed around 2*θ* = 25°, indicating that the carbon support possesses an amorphous or low-crystallinity structure. Additionally, peaks observed at approximately 2*θ* = 37, 43, 62, 74, and 78° were assigned to the (111), (200), (222), (311), and (222) reflections of cubic WC_1−*x*_ (ICCD PDF number: 00-020-1316, space group: *Fm*3̄*m*), respectively,^27^ which is in good agreement with the TEM observations. The value of *x* in the WC_1−*x*_ phase was estimated from the lattice constant calculated from the XRD patterns. The obtained lattice constant (approx. 4.24 Å) suggests that the stoichiometry of the synthesized tungsten carbide is approximately WC_0.5−0.7_ (*x* = 0.3–0.5) (see Section S2 in the SI for detailed calculation procedures), which is a typical non-stoichiometric phase formed under the rapid cooling conditions of the solution plasma process. As shown in Fig. S4, the Raman spectra for all carbon samples exhibit two prominent peaks: the G band at approximately 1600 cm^−1^ and the D band around 1350 cm^−1^. The G band corresponds to the sp^2^-hybridized carbon stretching modes, while the D band is associated with structural defects. Consequently, the intensity ratio (*I*_D_/*I*_G_) serves as a metric for the degree of graphitization and defect density.^28,29^ Notably, the *I*_D_/*I*_G_ ratio was found to decrease with increasing frequency, signifying an enhancement in the structural crystallinity of the carbon framework. This increased graphitization degree is expected to weaken the hydrogen bonding with the hydroxyl groups of cellulose, thereby decreasing the affinity between the carbon material and the cellulose fibers, because this structural evolution leads to a reduction in the number of active sites capable of forming hydrogen bonds with the hydroxyl (–OH) groups of cellulose due to a reduction in the number of active sites capable of forming hydrogen bonds with the hydroxyl (–OH) groups of cellulose.

**Table 3:** Tungsten contents of samples synthesized at different frequencies

Sample name	W [wt%]	C, O, H, *etc.* [wt%]
Benzen_W_12.5	4.03	96.97
Benzen_W_25.0	3.69	96.31
Benzen_W_37.5	2.92	97.08
Benzen_W_50.0	2.97	97.02

### 3.3 Surface chemical state and pore properties

**Fig. 5:**
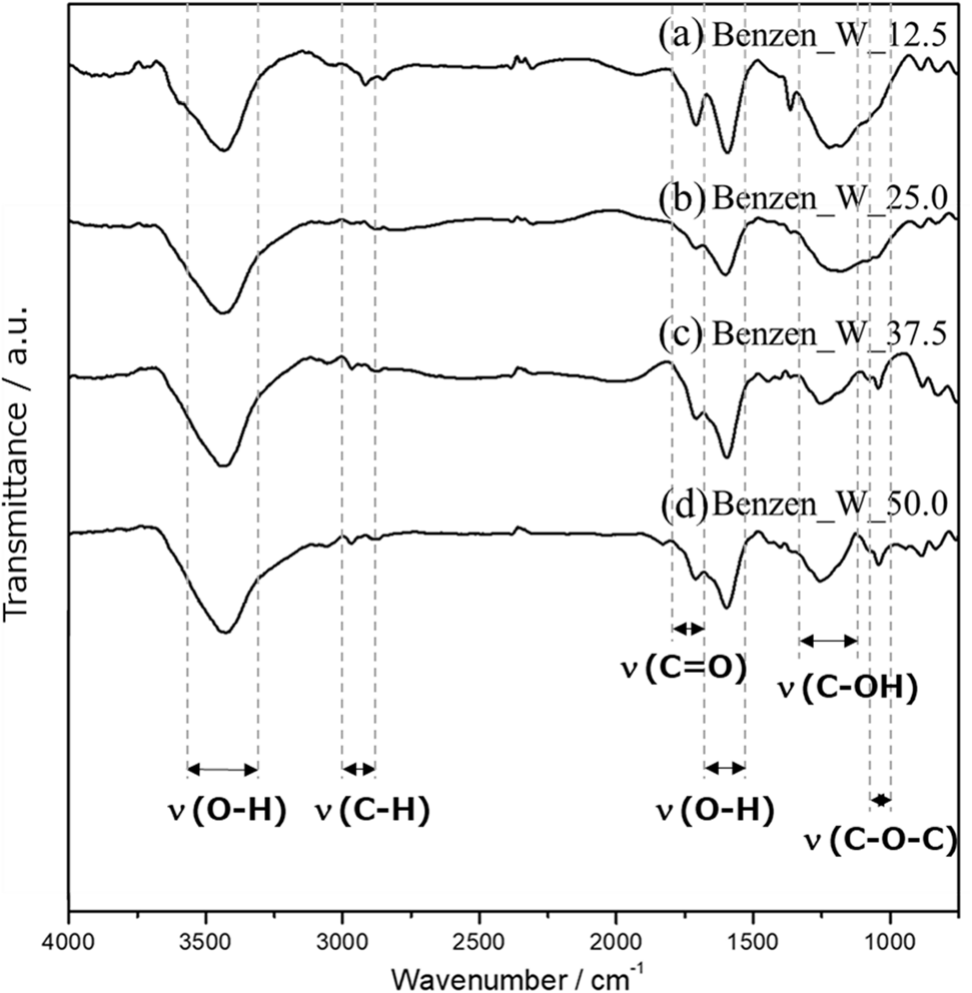
FT-IR spectra of samples synthesized at (a) 12.5, (b) 25.0, (c) 37.5, and (d) 50.0 kHz.

The surface functional groups of the synthesized samples were evaluated by FT-IR (Fig. 5). Absorption bands were observed around 1050, 1250, 1600, 1720, 2900, and 3400 cm^−1^, which were assigned to C–O–C, C–OH, O–H, C=O, and C–H bonds, respectively.^17,30^ In particular, the presence of hydroxyl (OH) groups is known to be a crucial factor that enhances affinity with hydrophilic cellulose molecules and promotes adsorption onto the catalyst surface.^2^ The chemical bonding states of the constituent elements were analyzed in detail using XPS (Fig. S5). For comparison, spectra of commercial WO_3_ and WC samples are also presented. In the W 4f spectrum of the synthesized sample (Fig. S5(a)), although the signal from W on the surface was extremely weak, the presence of tungsten carbide and some oxygen-related peaks was indicated. These oxygen species likely arise from the surface adsorption or oxidation during post-synthesis air exposure, as well as potential trace oxygen in the SP process. Although the presence of minor surface tungsten oxides (WO_*x*_) is possible, the core–shell structure can protect the internal WC_1−*x*_ from oxidation, maintaining its catalytic activity. This result corroborates the core–shell structure observed in the TEM images (Fig. 3), where the active WC_1−*x*_ core is encapsulated within thin carbon layers. Although a depth-resolved analysis was not performed, the combination of XRD and XPS results suggests that the detected oxygen species are primarily limited to the surface of the nanoparticles. The absence of WO_3_ diffraction peaks in the XRD patterns indicates that the WO_*x*_ species detected by XPS do not exist as a bulk phase but rather as a thin surface oxide layer or surface-adsorbed oxygen, which is partially protected by the surrounding carbon matrix. In contrast, the commercial WC reagent (Fig. S5(c)) showed strong peaks derived from oxidized tungsten (WO_3_). This suggests that the carbon shell formed by this method may play a protective role, preventing the atmospheric oxidation of the internal active species (WC_1−*x*_), a phenomenon consistent with the enhanced stability reported for carbon-encapsulated metal nanoparticles.^31^ However, it should be noted that the carbon shell encapsulating the WC_1−*x*_ nanoparticles is not an impermeable barrier. As suggested by the XRD, Raman spectroscopy, and FT-IR results, the carbon matrix possesses a disordered structure with numerous structural defects and micropores. These features allow water-soluble intermediates, such as glucose and cello-oligomers formed during the initial hydrothermal hydrolysis of cellulose, to diffuse through the thin carbon layers and reach the active tungsten carbide surfaces. Therefore, the core–shell structure provides a balance between protecting the active phase from deep oxidation and maintaining accessibility for the reactant intermediates. Furthermore, the C 1s spectra (Fig S5(b)) confirmed a trend where the proportion of C–O and C=O bonds decreased with increasing frequency. While the carbon matrix acts as a protective layer for the WC_1−*x*_ nanoparticles, the presence of surface oxygen species detected by FTIR and XPS suggests that minor surface oxidation occurs. These oxygen species are likely originated from atmospheric exposure during the post-synthesis handling and drying processes, rather than the synthesis itself, as the solution plasma was performed in an oxygen-free benzene solvent. It is important to note that the carbon shell effectively limits this oxidation to the surface, preventing the bulk transformation of WC_1−*x*_ into WO_3_, thereby preserving the intrinsic catalytic activity of the carbide phase.

Regarding the specific surface area and pore distribution (Fig. S6), all samples exhibited Type IV isotherms according to the IUPAC classification, confirming the presence of mesopores. The hysteresis loops observed in the high relative pressure region (*P*/*P*_0_ > 0.8) suggest the presence of macropores due to interparticle voids. The BET specific surface areas ranged from 196.0 to 236.6 m^2^ g^−1^, with the sample prepared at 12.5 kHz showing the maximum value. The relatively small difference in specific surface area is consistent with the SEM observations (Fig. S1), which showed no substantial changes in particle morphology or size among the samples. This relatively large surface area and the mesoporous structure of the WC_1−*x*_/C catalyst play a crucial role in the reaction efficiency. While the carbon shell provides a protective environment for the WC_1−*x*_ nanoparticles, the transport of bulky cellulose macromolecules to the internal active sites is generally limited. It is more plausible that the catalytic process involves the initial hydrolysis of cellulose into smaller, soluble intermediates, such as glucose and cello-oligomers, at the exterior surface of the catalyst. The relatively large surface area and mesoporous structure of the carbon matrix enhance the solid–solid contact and facilitate the transport of these soluble intermediates. These smaller molecules can then diffuse through structural defects or micropores in the carbon shell or interact with tungsten species in regions where the shell is sufficiently thin, subsequently undergoing the retro-aldol reaction to form lactic acid.

### 3.4 Catalytic performance for cellulose hydrothermal conversion

**Fig. 6:**
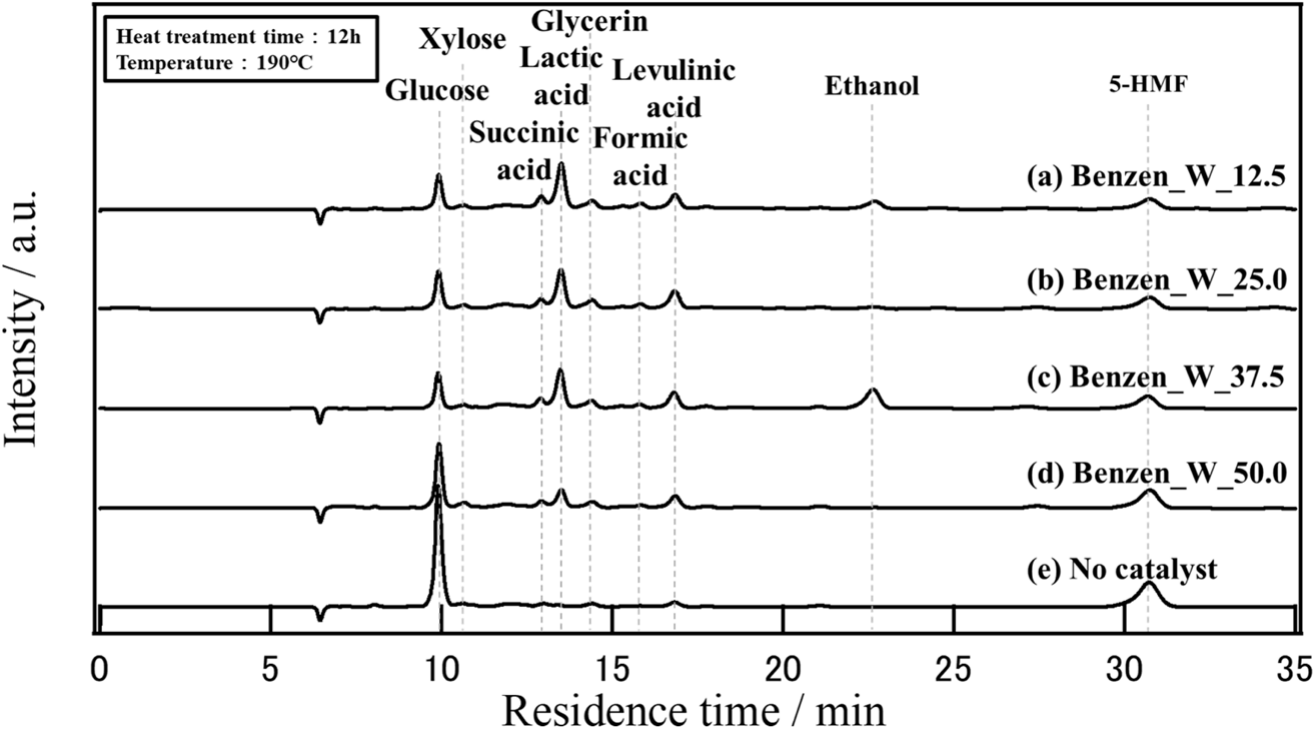
HPLC spectra of samples synthesized at (a) 12.5, (b) 25.0, (c) 37.5, and (d) 50.0 kHz, and (e) without catalyst.

**Fig. 7:**
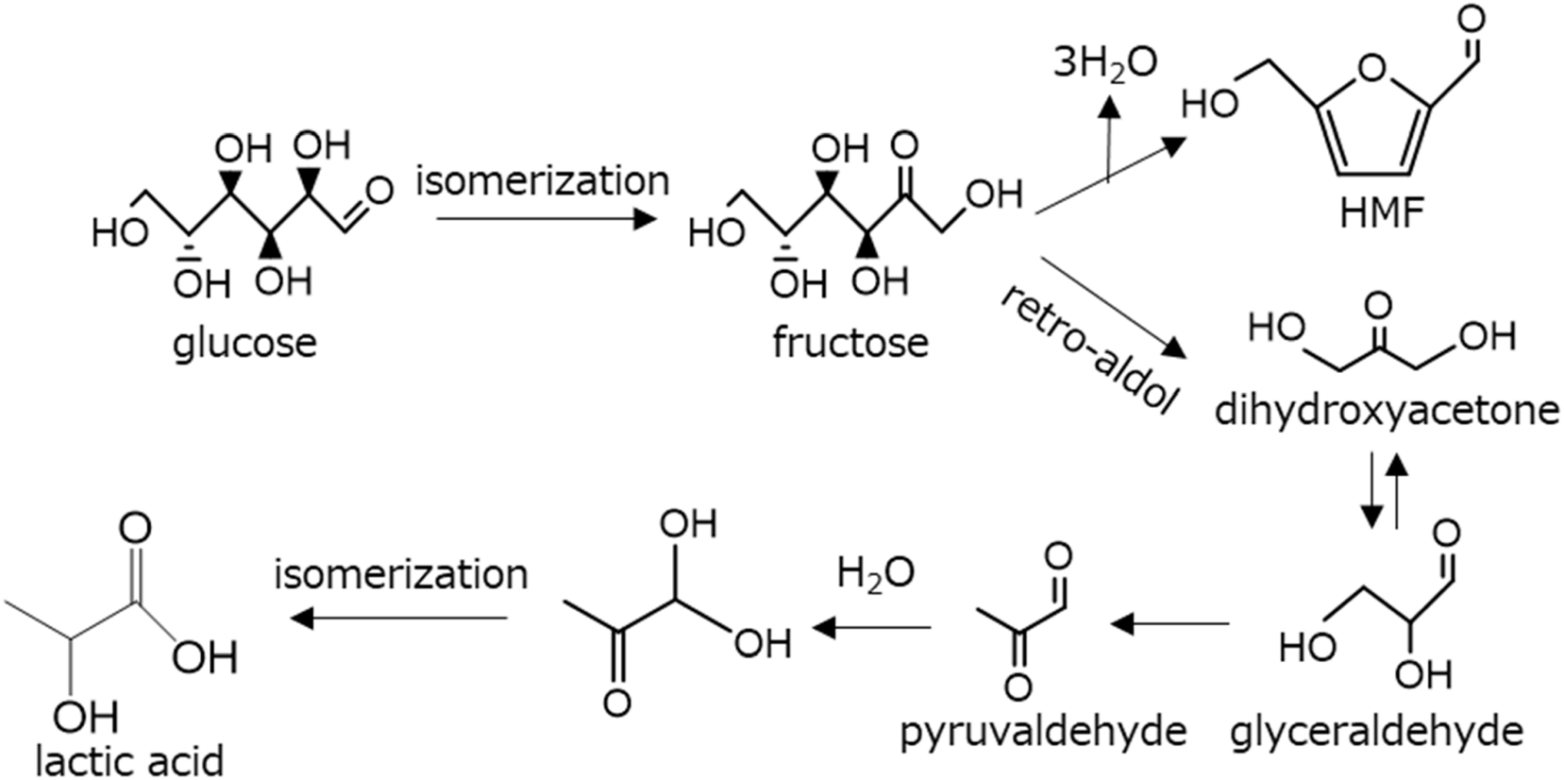
Schematic illustration of the formation mechanism of lactic acid from glucose in thermal hydrolysis tests.

Fig. 6 shows the product distribution (HPLC charts) from the cellulose hydrothermal conversion reaction using the synthesized catalysts. Glucose, lactic acid, organic acids (levulinic acid, formic acid), and 5-hydroxymethylfurfural (5-HMF) were detected as the main products. The use of catalysts with higher W content resulted in decreased glucose peak intensity and increased lactic acid production, suggesting that the supported WC_1−*x*_ nanoparticles catalyze the conversion of glucose to lactic acid. Based on the detected products, the reaction scheme is inferred *via* a multi-step pathway as follows (Fig. 7): first, cellulose undergoes hydrothermal hydrolysis to glucose, followed by isomerization to fructose. The high yield of lactic acid observed in this study provides indirect yet strong evidence for the occurrence of retro-aldol condensation of the fructose intermediate, which is known to be catalyzed by Lewis acid sites or tungsten-based species. The WC_1−*x*_ sites are responsible for the conversion of glucose intermediates to lactic acid. The reaction is driven by a combination of Lewis and Brønsted acidity. The WC_1−*x*_ nanoparticles act as Lewis acid sites that promote the isomerization and C–C bond cleavage of sugars. Brønsted acidity, provided by the surface functional groups on the carbon shell and the hydrothermal water, as well as the surface-coexisting WO_*x*_ species that can generate Brønsted acid sites (such as W–OH groups) under hydrothermal conditions,^32^ assists in the hydrolysis of cellulose. This interpretation aligns well with the reported bifunctional behavior of W_*x*_C–WO_3_ composite systems, where the synergistic cooperation between carbide and oxide phases facilitates both the initial hydrolysis of biomass and subsequent downstream conversions.^32^ The increase in pulse frequency, which results in higher W loading, likely increases the total number of Lewis acid sites, thereby accelerating the conversion of intermediates to lactic acid. This pathway is consistent with previous reports on tungsten carbide catalysts.^33,34^ The unique environment provided by the core–shell structure is thought to stabilize these intermediates and direct the reaction toward lactic acid rather than degradation products. The fact that lactic acid was obtained as the major product in this system suggests that the WC_1−*x*_ species functioned as Lewis acid sites that selectively promoted the retro-aldol reaction. It has been proposed that tungsten species may form tungsten bronze (H_*x*_WO_3_) intermediates under hydrothermal conditions, which serve as the intrinsic active sites for C–C bond cleavage.^31^ A question arises regarding the accessibility of cellulose-derived intermediates to the WC active sites, given the core–shell structure. Although the current study focuses on the overall conversion of cellulose, the observed high selectivity toward lactic acid strongly supports a reaction pathway involving the isomerization of glucose to fructose followed by retro-aldol condensation. It is widely recognized that the conversion of cellulose to lactic acid proceeds *via* the hydrothermal hydrolysis to glucose, followed by glucose-to-fructose isomerization and subsequent retro-aldol condensation of fructose.^35,36^ This mechanism is in excellent agreement with previously reported tungsten-based catalytic systems, where W species facilitate the specific cleavage of C3–C4 bonds in the keto-intermediate.^35,36^ Although TEM images confirm carbon encapsulation, the carbon layers formed by SP likely possess structural defects, micropores that allow reactant molecules to interact with the tungsten species or are sufficiently thin to allow reactant molecules to interact with the tungsten species. Additionally, the carbon shell may facilitate the adsorption of cellulose *via* hydrogen bonding between the oxygen-containing groups on the carbon surface and the hydroxyl groups of cellulose. The enhanced affinity of the carbon matrix for cellulose is attributed to the presence of oxygen-containing functional groups, such as hydroxyl and carboxyl groups, on the carbon surface, as confirmed by the FT-IR results (Fig. 5). These functional groups facilitate the formation of hydrogen bonds with the hydroxyl groups of the cellulose polymer, thereby promoting solid–solid interfacial contact and improving the adsorption of cellulose onto the catalyst surface.^37^

Furthermore, as proposed by Zhang *et al.*, tungsten species may undergo *in situ* structural transformation to form active tungsten bronze (H_*x*_WO_3_) species under hydrothermal conditions,^31^ which could facilitate the retro-aldol reaction *via* these accessible sites.^38^ The structural transformation from the bulk WC_1−*x*_ phase to the active H_*x*_WO_3_ species under hydrothermal conditions can be explained by the presence of a surface oxide layer and the hydrogen spillover effect. As evidenced by the XPS analysis (Fig. 4), the surface of the synthesized nanoparticles is partially oxidized, existing as WO_*x*_ species. During the hydrothermal reaction at 190 °C, hydrogen species generated from the dissociation of water or the initial decomposition of cellulose on the WC_1−*x*_ surface can migrate to the adjacent WO_*x*_ layer.^39,40^ This ‘hydrogen spillover’ leads to the partial reduction of WO_*x*_ and the subsequent formation of tungsten bronze (H_*x*_WO_3_), which serves as the intrinsic Lewis acid site for the retro-aldol reaction. In this synergistic mechanism, the WC_1−*x*_ core plays a dual role: providing a stable conductive support and acting as a promoter for hydrogen activation to maintain the H_*x*_WO_3_ phase.

**Fig. 8:**
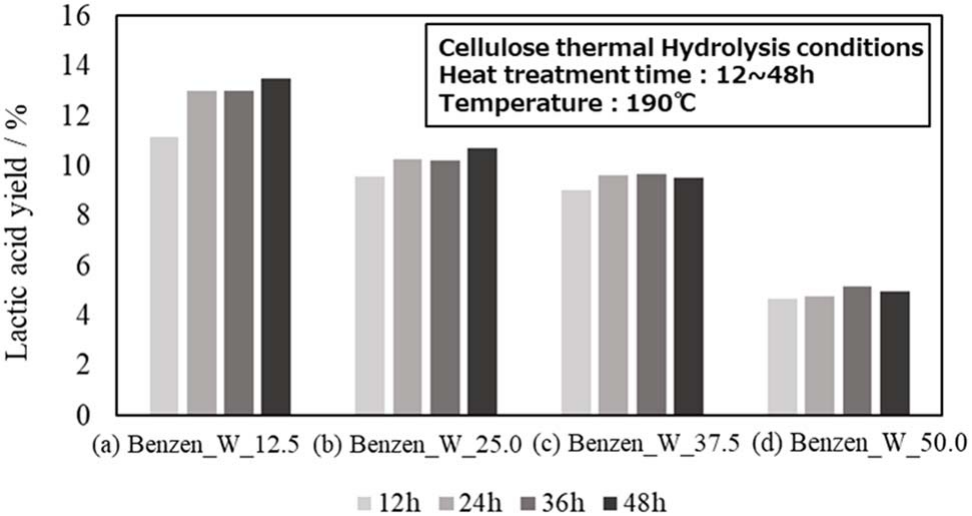
Lactic acid yield after cellulose thermal hydrolysis test using WC_1−*x*_ nanoparticle dispersed carbon catalysts synthesized at (a) 12.5, (b) 25.0, (c) 37.5, and (d) 50.0 kHz.

Fig. 8 shows the relationship between W content and lactic acid yield. The lactic acid yield improved with increasing W content, achieving a maximum yield of approximately 14.0% when using Benzene_W_12.5 (highest W content) under conditions of 190 °C for 48 h. To further investigate the influence of tungsten loading on the catalytic activity, the relationship between the W content and the lactic acid yield was plotted, as shown in Fig. S7. A clear positive correlation was observed, where the lactic acid yield increased as the W content rose from 2.9 to 4.0 wt%. This result suggests that the total number of tungsten species—specifically the highly dispersed WC_1−*x*_ and WO_*x*_ nanoparticles—acts as the primary factor determining the overall yield. The high dispersion of these active sites, achieved through the solution plasma process, facilitates the retro-aldol reaction of glucose, which is the rate-determining step in the conversion of cellulose to lactic acid. Furthermore, the stability of these active sites was confirmed by ICP-OES analysis of the filtrate, which showed negligible W leaching (less than 2 ppm), and the consistency of W content was supported by EDS mapping (Fig. S2 and Table S2) and CHN analysis (Table S1).

**Fig. 9:**
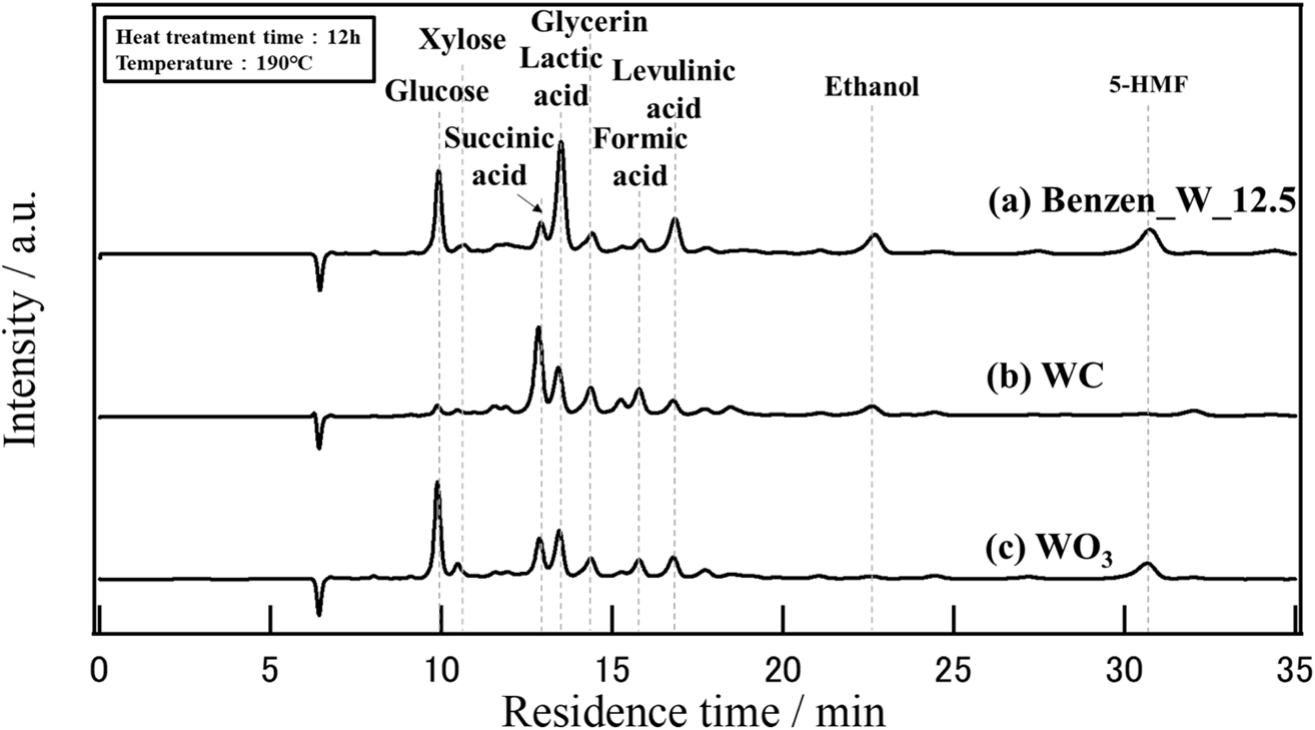
HPLC spectra of (a) benzene_W_12.5, (b) commercial WC, and (c) commercial WO_3_.

To verify the superiority of this catalyst, a comparison with commercial tungsten reagents was performed (Fig. 9). The lactic acid yields using commercial WO_3_ and WC remained at 5.1% and 5.3%, respectively. For an accurate comparison, we evaluated the intrinsic activity by considering the specific surface areas of these commercial samples. BET specific surface area of the obtained commercial WO_3_ and WC fine particles, which was determined by nitrogen-adsorption at 77 K, was found to be *ca.* 2.4 and 1.2 m^2^ g^−1^, respectively. Since the commercial bulk powders possess low surface areas, the WC_1−*x*_/C catalyst (SSA: 196.0–236.6 m^2^ g^−1^) provides a much higher density of active sites. This structural advantage, characterized by the highly dispersed nanocrystalline WC_1−*x*_ phases within the porous carbon matrix, fundamentally contributes to its superior catalytic performance compared to commercial bulk phases. This enhancement is primarily attributed to the increased specific surface area achieved through the fine nanoparticles formed *via* the SP method, alongside the dispersion stabilization effect provided by the carbon support, which prevents the aggregation of active sites.

**Fig. 10:**
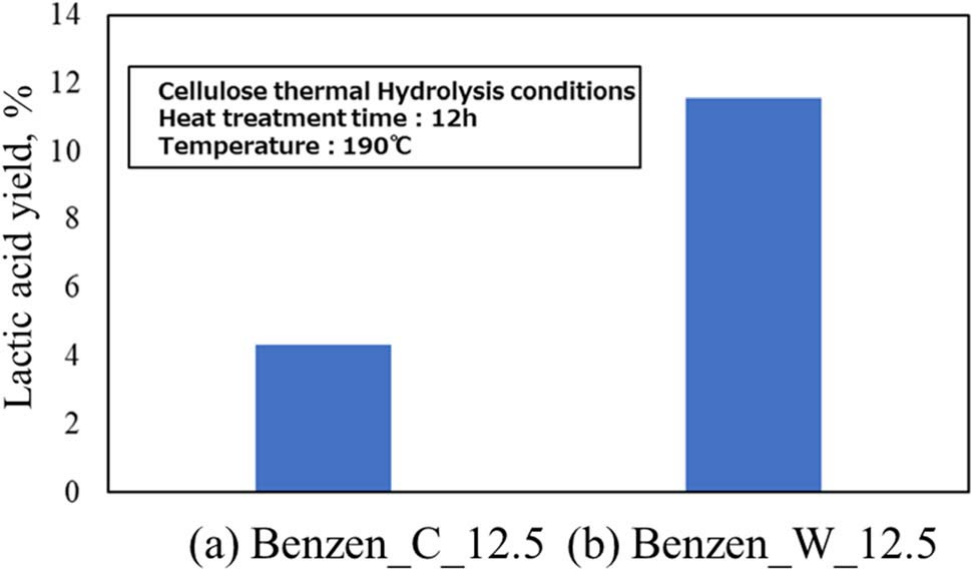
Lactic acid yield after cellulose thermal hydrolysis test using carbon catalysts synthesized by solution plasma at 12.5 kHz using (a) carbon fiber and (b) tungsten electrodes.

Furthermore, compared to previous reports (Table 4), although the yield is lower than that of AlW or ZrW systems, the present catalyst exhibits a superior yield compared to solid acid catalysts such as ZrS and sulfonated carbon (C-SO_3_H). Finally, to prove that the active sites originate from WC_1−*x*_, a control experiment was conducted using a tungsten-free carbon sample (Benzene_C_12.5) synthesized using carbon fiber electrodes. EDS analysis confirmed the absence of W in this carbon sample (the corresponding data has been shown in Fig. S8). The results clearly confirm the absence of tungsten signals, ensuring that the catalytic activity observed in WC sample is solely attributed to the introduced tungsten carbide species. When this W-free sample was used, the lactic acid yield remained at 4.3% (Fig. 10). In contrast, the yield for Benzene_W_12.5 under the same conditions (190 °C, 12 h) was 11.5%. Although the carbon support itself shows slight activity due to adsorption effects by functional groups, this experiment unequivocally demonstrated that the presence of WC_1−*x*_ nanoparticles is essential for the dramatic improvement in yield. To verify the heterogeneous nature of the catalysis, the concentration of leached tungsten species in the reaction filtrate was analyzed by ICP-OES (the details of the estimation method are shown in SI as S1). The result showed that the amount of dissolved tungsten was negligible (<2 ppm), confirming that the reaction proceeds on the solid catalyst surface and that the WC nanoparticles are stably encapsulated within the carbon matrix during the hydrothermal process.

**Table 4:** Comparison of catalytic performances for the conversion of cellulose to lactic acid over various catalysts

Substrate	Catalyst	Solvent	*T* (K)	*t* (h)	Yield (%)	Ref.
Cellulose	C-SO_3_H	Water	463	24	4^a^	17
Cellulose	ZrS	Water	463	24	3	31
Cellulose	AlW	Water	463	24	28	16
Cellulose	ZrW	Water	463	24	19	16
Cellulose	Nb@AlF_3_	Water	453	2	27	2
Cellulose	ZrO_2_	Water	473	6	21	41
Cellulose	ZrO_2_-Al_2_O_3_	Water	473	6	25	42
Cellulose	Benzen_W_12.5	Water	463	12	12	This study
Cellulose	Benzen_W_12.5	Water	463	24	13	This study
Cellulose	Benzen_W_12.5	Water	463	36	13	This study
Cellulose	Benzen_W_12.5	Water	463	48	14	This study

a Yield of glucose. (The other catalyst yields represent lactic acid).

To further evaluate the catalytic stability and reusability, recycling tests were conducted using the recovered WC_1−*x*_/C catalyst synthesized at 12.5 kHz. As summarized in Table 5, the lactic acid yield exhibited a significant decrease from 13.98% in the first cycle to 5.08% in the second cycle, eventually reaching 3.67% by the fourth cycle. Notably, the yield in the fourth cycle fell slightly below that of the carbon support alone (4.31%). While the ICP-OES analysis of the filtrate after the first cycle showed negligible tungsten leaching (<2 ppm), suggesting an initially stable heterogeneous system, the subsequent decline in activity points to a gradual structural degradation of the catalyst.

**Table 5:** Reusability of WC_1−*x*_/C catalyst for the conversion of cellulose to lactic acid over four cycles

Catalytic cycle	Lactic acid yield (%)
1st cycle	13.98
2nd cycle	5.08
3rd cycle	4.92
4th cycle	3.67
Reference: carbon support only	4.31

To investigate this deactivation, the spent catalyst after four cycles was characterized by SEM, EDS, and XRD (Fig. S9–S11). The XRD analysis (Fig. S11) revealed that the characteristic peaks of the WC_1−*x*_ phase, which were clearly present in the fresh catalyst, completely disappeared after the repeated hydrothermal treatments. This indicates that the active carbide species underwent a loss of crystallinity or phase transformation under the harsh reaction conditions (190 °C). Furthermore, SEM observations (Fig. S9) confirmed a distinct change in the surface morphology. The fact that the final yield was lower than that of the carbon support alone may be attributed to the deposition of these degraded or amorphous tungsten species on the carbon surface, potentially blocking the inherent active sites or porous structure of the carbon matrix. These results demonstrate that while the carbon shell initially prevents metal leaching, the structural robustness of the WC_1−*x*_ core needs further improvement to maintain catalytic performance over multiple cycles.

## 4. Conclusions

In this study, carbon composite materials featuring highly dispersed tungsten carbide (WC_1−*x*_) nanoparticles (WC_1−*x*_/C) were successfully synthesized *via* a rapid, one-pot solution plasma (SP) process. Structural analyses revealed that the synthesized nanoparticles were cubic WC_1−*x*_ crystals possessing a core–shell structure encapsulated by several layers of graphene shells. The pulse frequency applied during the SP process proved to be a critical factor in controlling the product composition; specifically, lowering the frequency effectively increased the tungsten content within the carbon matrix. Based on the hydrothermal conversion of cellulose using the synthesized WC_1−*x*_/C catalysts, the following conclusions were drawn:

(1) A positive correlation was observed between the lactic acid yield and the tungsten content in the catalyst. This demonstrates that the tungsten species function as active sites for the conversion of cellulose to lactic acid, particularly by promoting the retro-aldol reaction.

(2) The catalyst with the highest tungsten loading (synthesized at 12.5 kHz) achieved a maximum lactic acid yield of approximately 14.0% under reaction conditions of 190 °C for 48 h. This performance was significantly higher than that obtained using a tungsten-free carbon sample synthesized with carbon fiber electrodes (4.3% yield) or commercial WC and WO_3_ reagents.

(3) Reusability tests and post-reaction characterizations revealed that while the graphene shell effectively minimized tungsten leaching after the first cycle (<2 ppm), the catalytic activity decreased over consecutive cycles. This deactivation was attributed to the loss of crystallinity and structural degradation of the WC_1−*x*_ phase under the harsh hydrothermal conditions, as confirmed by XRD and SEM analyses.

These results highlight the superior initial catalytic performance arising from the unique nanocomposite structure obtained *via* the SP process. Although further improvement in the structural robustness of the carbide phase is required for long-term stability, the SP-based synthesis strategy proposed herein is an environmentally benign process that eliminates the need for high-temperature calcination or reducing gases. Consequently, this method holds great promise for the development of new functional catalysts for the valorization of biomass resources.

## Author contributions

Conceptualization and methodology, T. I.; experimental and data analysis, K. Y., U. A., A. U., T. I., T. I., and G. P.; writing—original draft preparation, K. Y. and T. I.; writing—review and editing, T. I.; supervision, T. I.; project administration and funding acquisition, T. I. All authors have read and agreed to the published version of the manuscript.

## Conflicts of interest

There are no conflicts to declare.

## Supplementary Material

RA-016-D5RA09791F-s001

## Data Availability

The data supporting this article have been included as part of the supplementary information (SI). Supplementary information: estimation of the dissolved metal ion concentration by ICP-OES, estimation of the *x* value in the WC_1−*x*_ phase, contents (wt%) of elemental C, H, and O in the synthesized carbon samples, quantitative EDS analysis of the synthesized samples, FE-SEM images, elemental mapping image, particle size distribution histogram, Raman spectra, XPS spectra, N_2_ adsorption–desorption isotherms, relationship between W content (wt%) in tungsten carbide nanoparticle-dispersed carbon catalysts and lactic acid yield (%) after hydrothermal conversion of cellulose, SEM image and EDS spectrum of the WC sample synthesized by SPP using a C electrode, SEM images of the WC_1−*x*_/C catalyst before and after catalytic cycles, EDS analysis of the WC_1−*x*_/C catalyst before and after catalytic cycles, and XRD patterns of the WC_1−*x*_/C catalyst before and after four catalytic cycles. See DOI: https://doi.org/10.1039/d5ra09791f.

## References

[cit1] Lashof D. A., Ahuja D. R. (1990). Relative contributions of greenhouse gas emissions to global warming. Nature.

[cit2] Wang L., Zhang Y., Peng L., Li H., Xue Y. (2017). Catalytic conversion of cellulose to lactic acid over Nb-doped AlF_3_ catalysts with balanced acidity. Catal. Commun..

[cit3] Huber G. W., Iborra S., Corma A. (2006). Synthesis of Transportation Fuels from Biomass: Chemistry, Catalysts, and Engineering. Chem. Rev..

[cit4] Chheda J. N., Huber G. W., Dumesic J. A. (2007). Liquid-Phase Catalytic Processing of Biomass-Derived Oxygenated Hydrocarbons to Fuels and Chemicals. Angew. Chem. Int. Ed..

[cit5] Li C., Zhao X., Wang A., Huber G. W., Zhang T. (2015). Catalytic Transformation of Lignin for the Production of Chemicals and Fuels. Chem. Rev..

[cit6] Zhang Z., Song J., Han B. (2017). Catalytic Transformation of Lignocellulose into Chemicals and Fuel Products in Ionic Liquids. Chem. Rev..

[cit7] Yabushita M., Kobayashi H., Fukuoka A. (2014). Catalytic transformation of cellulose into platform chemicals. Appl. Catal., B.

[cit8] Li S., Deng W., Li Y., Zhang Q., Wang Y. (2019). Catalytic conversion of cellulose-based biomass and glycerol to lactic acid. J. Energy Chem..

[cit9] Zartman W. H., Adkins H., of Sugars H. (1933). J. Am. Chem. Soc..

[cit10] Qi X., Watanabe M., Aida T. M., Smith Jr. R. L. (2008). Catalytical conversion of fructose and glucose into 5-hydroxymethylfurfural in hot compressed water by microwave heating. Catal. Commun..

[cit11] Yan H., Yang Y., Tong D., Xiang X., Hu C. (2009). Catalytic conversion of glucose to 5-hydroxymethylfurfural over SO_4_^2−^/ZrO_2_ and SO_4_^2−^/ZrO_2_–Al_2_O_3_ solid acid catalysts. Catal. Commun..

[cit12] Serrano-Ruiz J. C., West R. M., Dumesic J. A. (2010). Catalytic Conversion of Renewable Biomass Resources to Fuels and Chemicals. Annu. Rev. Chem. Biomol. Eng..

[cit13] Matsumoto K., Kobayashi H., Ikeda K., Komanoya T., Fukuoka A., Taguchi S. (2011). Chemo-microbial conversion of cellulose into polyhydroxybutyrate through ruthenium-catalyzed hydrolysis of cellulose into glucose. Bioresour. Technol..

[cit14] Komesu A., de Oliveira J. A. R., da Silva Martins L. H., Maciel M. R. W., Maciel Filho R. (2017). Lactic Acid Production to Purification: A Review. BioResources.

[cit15] Abdel-Rahman M. A., Tashiro Y., Sonomoto K. (2013). Recent advances in lactic acid production by microbial fermentation processes. Biotechnol. Adv..

[cit16] Chambon F., Rataboul F., Cabiac A., Guillon E., Essayem N. (2011). Cellulose hydrothermal conversion promoted by heterogeneous Brønsted and Lewis acids: Remarkable efficiency of solid Lewis acids to produce lactic acid. Appl. Catal., B.

[cit17] Suganuma S., Nakajima K., Kitano M., Yamaguchi D., Kato H., Hayashi S., Hara M. (2008). Hydrolysis of Cellulose by Amorphous Carbon Bearing SO3H, COOH, and OH Groups. J. Am. Chem. Soc..

[cit18] Demetriou M. D., Ghoniem N. M., Lavine A. S. (2002). Computation of metastable phases in tungsten-carbon system. J. Phase Equilib..

[cit19] Perera S. M. H. D., Ciuffetelli E., Porosoff M. D. (2026). Achieving Phase Control of Polymorphic Tungsten Carbide Catalysts. ACS Catal..

[cit20] Bretzler P., Huber M., Rane A. A., Jentoft R. E., Köhler K., Jentoft F. C. (2022). Selective synthesis of tungsten carbide phases W_2_C and WC as hydrogenation catalysts. J. Catal..

[cit21] Ma L., Wang W., Wang H., Zhang Y., Wang Y. (2018). Synthesis of tungsten carbide nanoparticles by a modified carburization process. J. Alloys Compd..

[cit22] Kang J., Li O. L., Saito N. (2013). Synthesis of structure-controlled carbon nano spheres by solution plasma process. Carbon.

[cit23] Janpetch N., Saito N., Rujiravanit R. (2016). Fabrication of bacterial cellulose-ZnO composite via solution plasma process for antibacterial applications. Carbohydr. Polym..

[cit24] Saito N., Hieda J., Takai O. (2009). Synthesis process of gold nanoparticles in solution plasma. Thin Solid Films.

[cit25] Kim D.-W., Li O. L., Pootawang P., Saito N. (2014). Solution plasma synthesis process of tungsten carbide on N-doped carbon nanocomposite with enhanced catalytic ORR activity and durability. RSC Adv..

[cit26] Boonyeun N., Rujiravanit R., Saito N. (2021). Deposition of carbon–tungsten carbide on coir pulp to improve its compatibility with polylactic acid. Cellulose.

[cit27] Ji N., Zhang T., Zheng M., Wang A., Wang H., Wang X., Chen J. G. (2008). Direct Catalytic Conversion of Cellulose into Ethylene Glycol Using Nickel-Promoted Tungsten Carbide Catalysts. Angew. Chem. Int. Ed..

[cit28] Ferrari A. C., Robertson J. (2000). Interpretation of Raman Spectra of Amorphous and Disordered Carbon. Phys. Rev. B:Condens. Matter Mater. Phys..

[cit29] Ferrari A. C. (2007). Raman Spectroscopy of Graphene and Graphite: Disorder, Electron–Phonon Coupling, Doping and Nonadiabatic Effects. Solid State Commun..

[cit30] Szabó T., Berkesi O., Dékány I. (2005). DRIFT Spectra of Deuterated Graphite Oxide. Carbon.

[cit31] Zheng M., Wang A., Ji N., Wang H., Huang C., Wang X., Zhang T. (2010). Transition Metal Carbides for the Direct Conversion of Cellulose into Chemicals. ChemSusChem.

[cit32] Liu Q., Zhang J., Wang H., Liu X., Zheng M., Zhang T. (2025). Bifunctional Behavior and Synergistic Mechanisms of Tungsten Carbide-Oxide Composites in Biomass Conversion. J. Am. Chem. Soc..

[cit33] Liu Y., Huang C., Wang T., Jiang L., Zheng M., Wang A., Zhang T. (2019). Selective Conversion of Cellulose into Lactic Acid over Carbon-Supported Tungsten Carbide Catalysts. ACS Catal..

[cit34] Karim A. M., Prasad V., Mpourmpakis G., Lonergan W. W., Frenkel A. I., Chen J. G., Vlachos D. G. (2009). Correlating Structure and Reactivity of Carbon-Supported Tungsten Carbide Catalysts for Water-Gas Shift and Hydrogenation Reactions. J. Am. Chem. Soc..

[cit35] Zheng M., Wang A., Ji N., Pang J., Wang X., Zhang T. (2010). Selective conversion of cellulose into ethanol and ethylene glycol: the synergistic action of tungsten carbide and carbon-supported ruthenium catalysts. Commun. Chem..

[cit36] Wang P., Zheng M., Wang A., Zhang T. (2021). Selective C3–C4 Bond Cleavage of Sugars over Tungsten-Based Catalysts. J. Energy Chem..

[cit37] Kobayashi H., Komanoya T., Hara K., Fukuoka A. (2010). Water-Tolerant Solid Acid Catalysts for the Hydrolysis of Cellulose. ChemSusChem.

[cit38] Ji N., Zheng M., Wang A., Zhang Y., Zhang T. (2012). Direct Catalytic Conversion of Cellulose into Base Chemicals: New Opportunities for Tungsten Carbide Catalysts. ChemSusChem.

[cit39] Ji J., Jiang L., Tang W., Wu S., Wang Z. (2022). Hydrogen Spillover on Tungsten Carbide/Oxide Heterostructure for Efficient Hydrolytic Hydrogenation of Biomass. ACS Catal..

[cit40] Yu Y., Li G., Wu S., Wang Z. (2020). Active Phase Transition and Hydrogen Spillover on Tungsten-Based Catalysts During Biomass Hydrolytic Hydrogenation. J. Catal..

[cit41] Yan P., Wang Y., Fu G., Jiao L., Zhang S. (2015). One-pot conversion of cellulose into lactic acid over a zirconia-based catalyst in water. RSC Adv..

[cit42] Kim J., Kim S., Kim E. S., Jae J., Kim M. Y. (2021). Effect of ZrO_2_-Al_2_O_3_ mixed oxide support on the catalytic performance of Sn-based catalysts for the conversion of cellulose to lactic acid. Mol. Catal..

