# Supplementary material for: One-pot solution plasma synthesis of tungsten carbide core–shell nanoparticles for efficient conversion of cellulose to lactic acid
Source: RSC Adv. 2026 Jul 2;16(35):35067–77. doi: 10.1039/d5ra09791f (PMC13326196; doi:10.1039/d5ra09791f)
Supplement: RA-016-D5RA09791F-s001 [file RA-016-D5RA09791F-s001.pdf]

Supplementary Information (SI) for RSC Advances.
This journal is © The Royal Society of Chemistry 2026

Electronic Supplementary Information

One-Pot Solution Plasma Synthesis of Carbon-Supported Tungsten Carbide Nanoparticles for Efficient Conversion of Cellulose to Lactic Acid

Kouki Yamamoto[1], Yuki Atsuumi[1], Akinari Uesugi[1], Taketo Imamura[1], Toshiki Iwato[1], Gasidit Panomsuwan[2], Takahiro Ishizaki[3]*

[1]Materials Science and Engineering, Graduate School of Engineering and Science, Shibaura Institute of Technology, Toyosu, Koto-Ku, Tokyo, 135-8548, Japan

[2]Department of Materials Engineering, Faculty of Engineering, Kasetsart University, Bangkok 10900, Thailand

[3]College of Engineering, Shibaura Institute of Technology, Toyosu, Koto-Ku, Tokyo, 135-8548, Japan

* E-mail：ishizaki@shibaura-it.ac.jp

S1 Estimation of the dissolved metal ion concentration by ICP-OES

The dissolved metal ion concentration in the mixed aqueous solution containing catalyst, pretreated cellulose, and ultrapure water after hydrothermal conversion was measured using an inductively coupled plasma optical emission spectrometer (5110, manufactured by Agilent Technologies Inc.; ICP-OES) and a calibration curve method. ICP mixed standard solution for quantitative analysis of the dissolved metal ion concentration. The concentration of leached tungsten in the reaction filtrate was quantified by ICP-OES using a multi-point calibration curve. A series of standard solutions (0, 1.0, 3.0, 5.0, 8.0, and 10.0 ppm) was prepared by diluting a certified tungsten standard solution (1000 mg/L) with ultrapure water. The emission line at 239.709 nm was used for tungsten detection. The calibration line showed excellent linearity with a correlation coefficient (R^2) exceeding 0.999. Before measurement, the reaction filtrate was filtered through a 0.1 μm membrane filter and acidified with 2% HNO_3 to ensure the stability of the metal ions. The analysis confirmed that the W concentration was consistently below 2 ppm across all samples.

S2 Estimation of the x value in the WC_{1-x} phase

The non-stoichiometric value x in the cubic WC_{1-x} phase was estimated based on the lattice constant (a) determined from the X-ray diffraction (XRD) patterns. For the cubic

structure (space group: $Fm\bar{3}m$), the lattice constant, a, was calculated using the interplanar spacing (d) of the prominent crystallographic planes according to the standard formula:

$$a = d \cdot \sqrt{h^2 + k^2 + l^2}$$

In this calculation, the experimental 2θ positions of the (111), (200), and (220) planes observed at approximately 37°, 43°, and 62°, respectively, were utilized. The calculated lattice constant of the synthesized nanoparticles was approximately 4.24 Å.

According to the literature data for the non-stoichiometric rock-salt type cubic tungsten carbide phase, the lattice constant linearly varies with the carbon content (Vegard's law), where a stoichiometric WC1.0 (or β-$WC_{1.0}$ possesses a lattice constant of approximately 4.27 Å, whereas carbon-deficient phases such as W_2C or $WC_{0.5}$ exhibit values around 4.22–4.23 Å. Therefore, the obtained lattice constant of ca. 4.24 Å corresponds to a chemical stoichiometry of approximately $WC_{0.5\text{-}0.7}$, which yields an estimated x value ranging from 0.3 to 0.5.

Table S1: Contents (wt.%) of elemental C, H, and O in the carbon samples synthesized at (a) 12.5, (b) 25.0, (c) 37.5, and (d) 50.0 kHz.

Frequency	C (wt.%)	H (wt.%)	O (wt.%)
(a) 12.5 (kHz)	88.77	2.57	6.63
(b) 25.0 (kHz)	88.97	1.45	5.89
(c) 37.5 (kHz)	90.13	1.43	5.52
(d) 50.0 (kHz)	91.45	1.42	4.15

Table S2 Quantitative EDS analysis of the samples synthesized at a frequency of (a) 12.5, (b) 25, (c) 37.5, and (d) 50 kHz corresponding to Figure S2.

Sample name	C (at.%)	O (at.%)	W (at.%)
(a) 12.5 (kHz)	95.64	4.30	0.06
(b) 25.0 (kHz)	94.54	5.38	0.08
(c) 37.5 (kHz)	91.10	7.50	1.40
(d) 50.0 (kHz)	94.58	5.34	0.08

Figure S1: FESEM images of the samples synthesized at a frequency of (a) 12.5, (b) 25, (c) 37.5, and (d) 50 kHz.

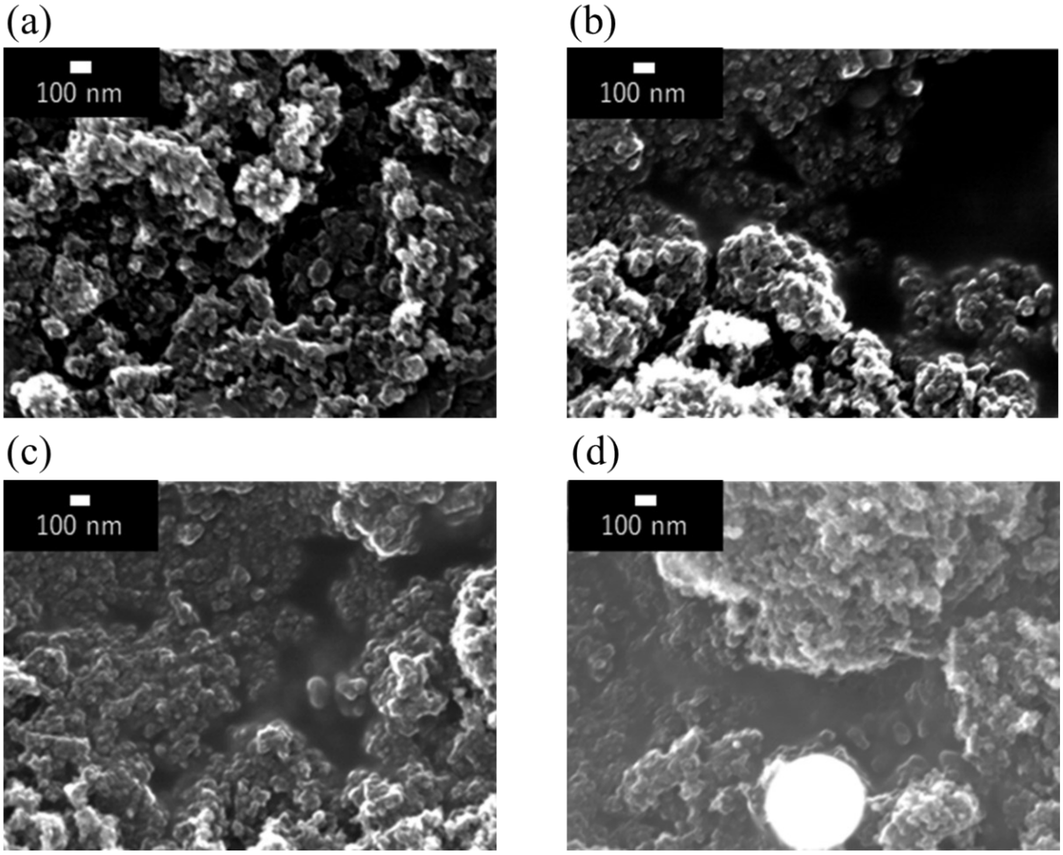

Figure S2. Elemental (C, O, and W) mapping images of the samples synthesized at a frequency of (a) 12.5, (b) 25, (c) 37.5, and (d) 50 kHz.

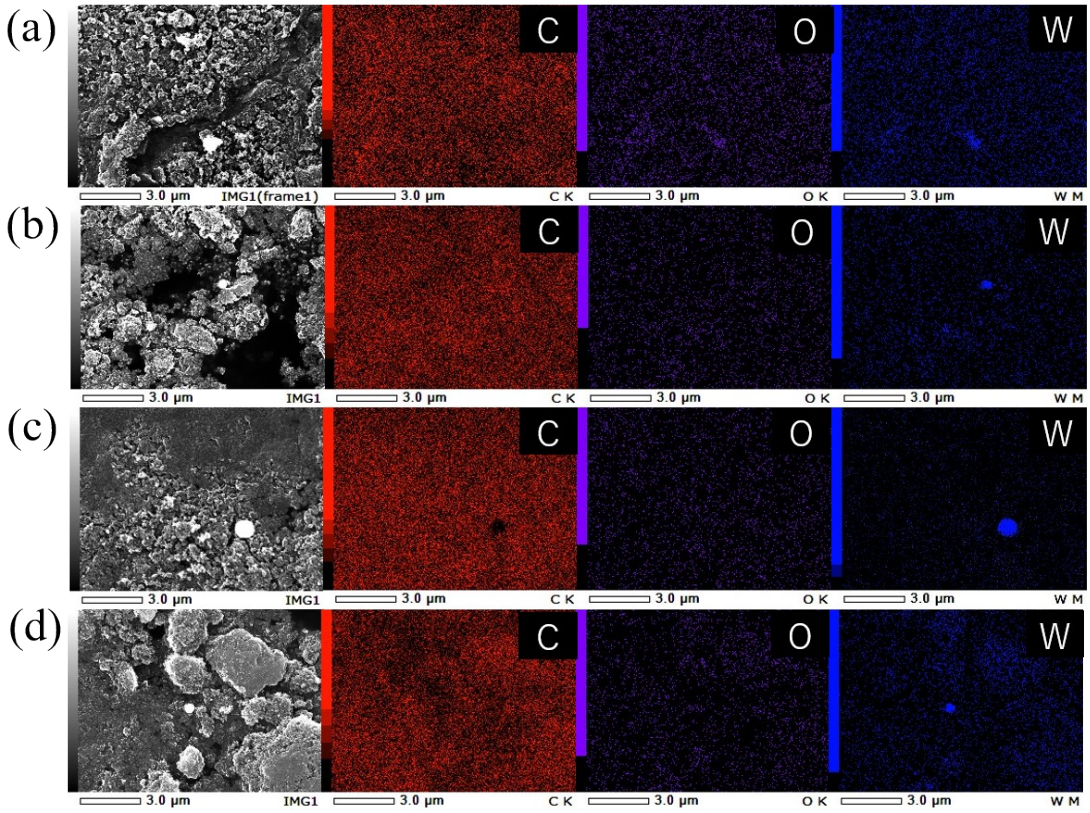

Figure S3: Particle size distribution histogram of the tungsten carbide nanoparticles synthesized at a frequency of 12.5 kHz.

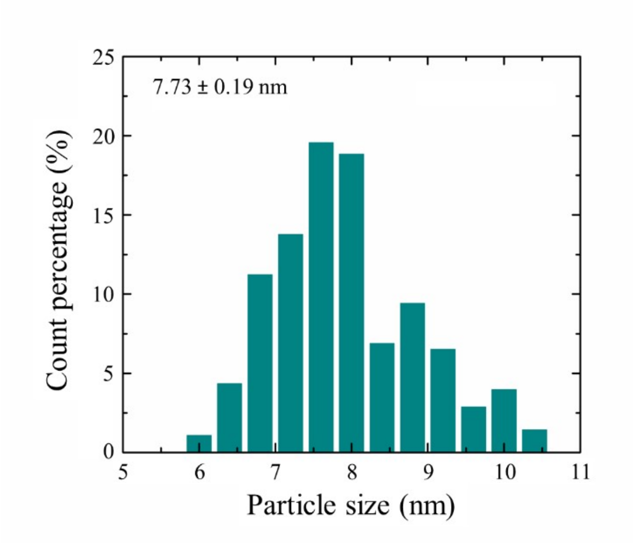

Figure S4: Raman spectra and the peak ratios I_D/I_G values of the synthesized carbon materials by solution plasma at frequencies of (a) 12.5, (b) 25.0, (c) 37.5, and (d) 50.0

kHz.

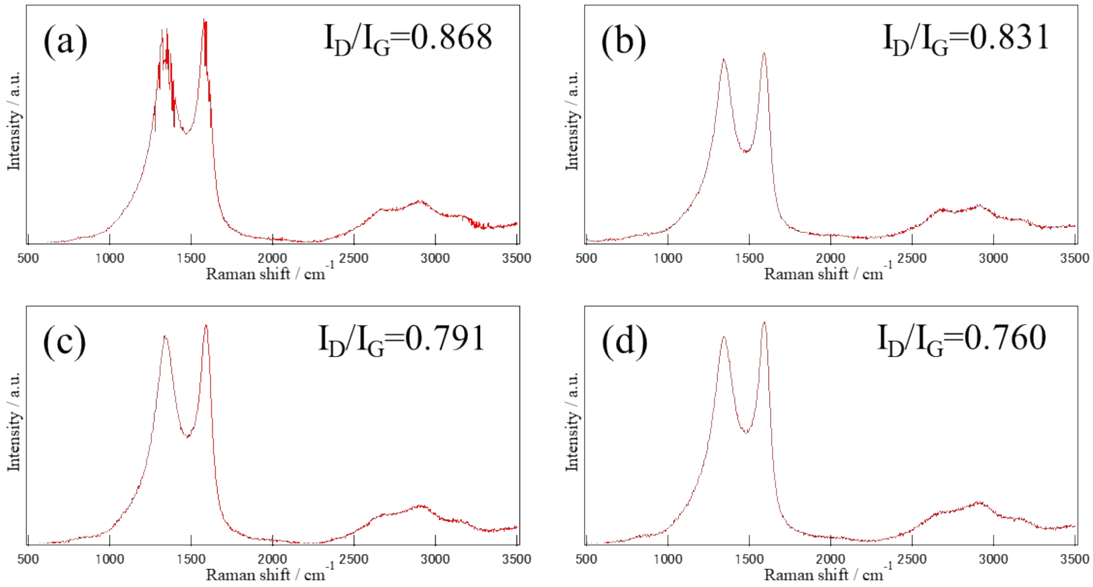

Figure S5: (a) W 4f, (b) C 1s, and (c) O 1s XPS spectra of the samples synthesized at a frequency of 12.5, 25, 37.5, and 50 kHz.

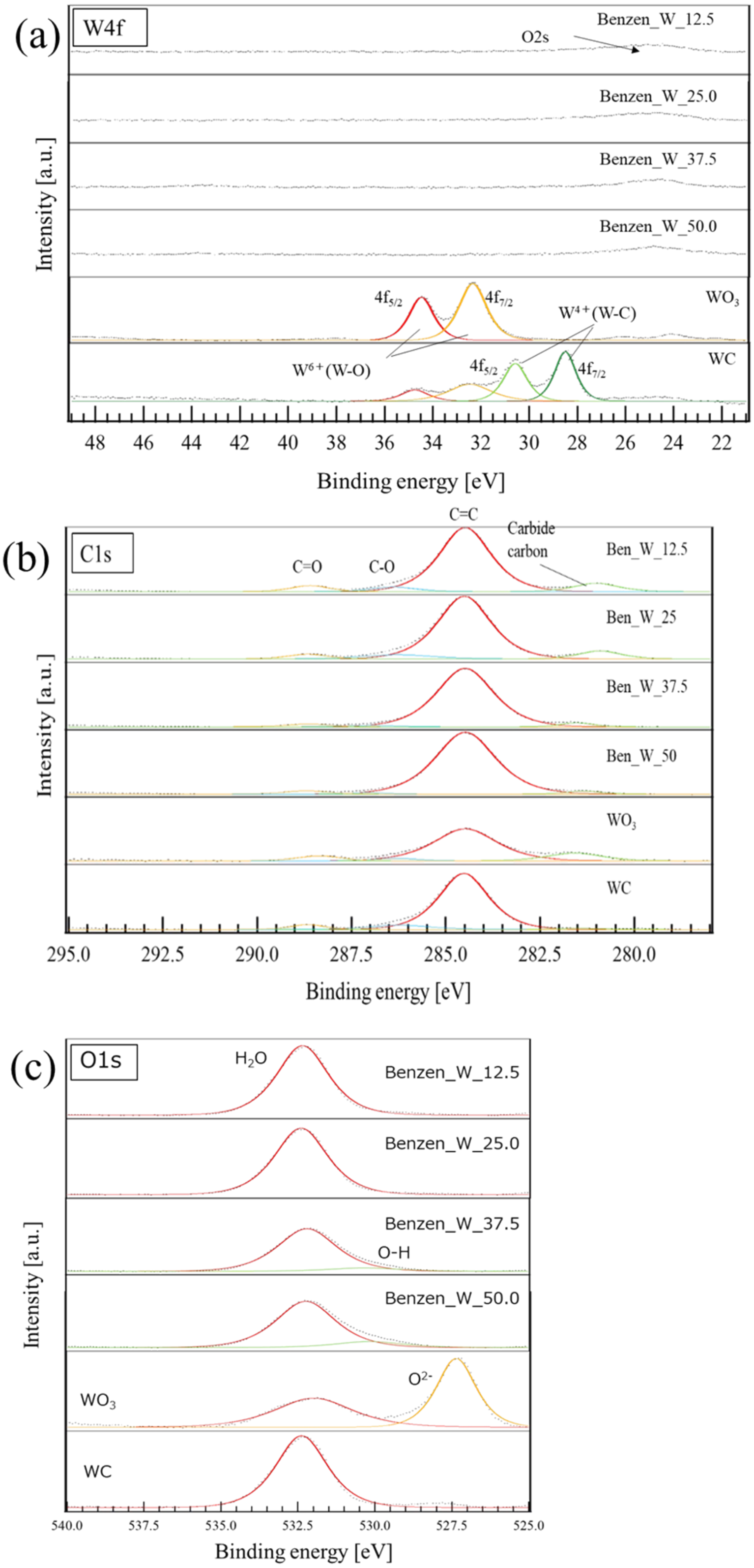

Figure S6: N_2 adsorption-desorption isotherms of the samples synthesized at a frequency of (a) 12.5, (b) 25, (c) 37.5, and (d) 50 kHz.

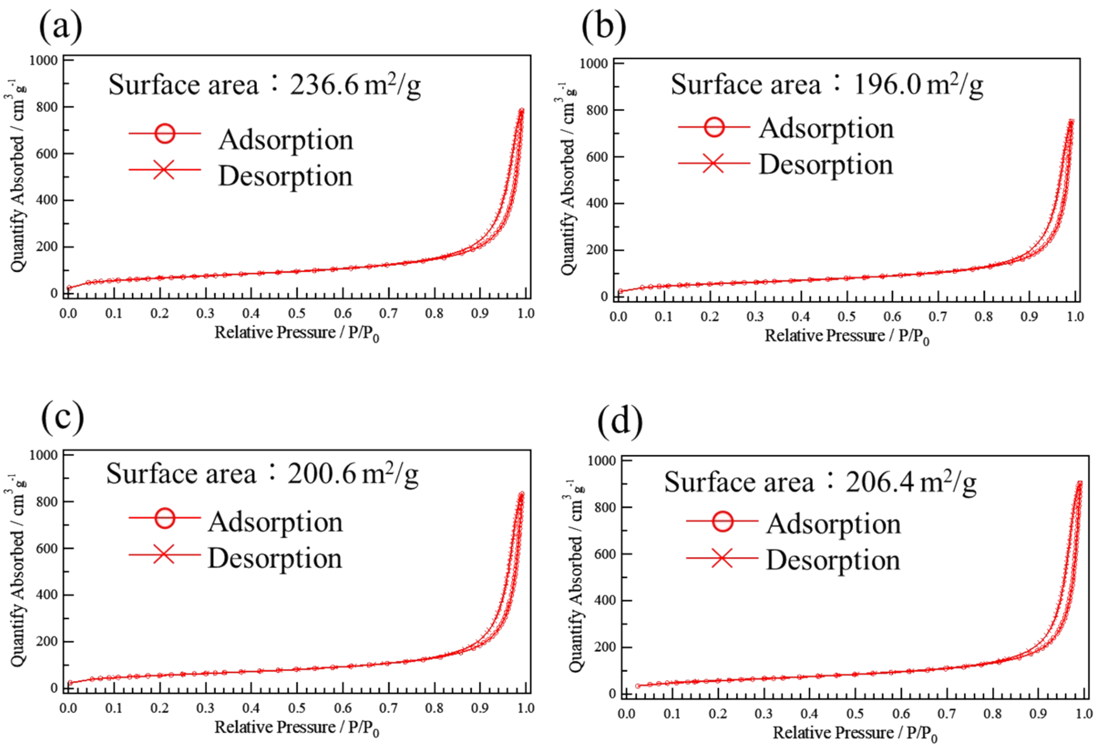

Figure S7: Relationship between W content (wt.%) in tungsten carbide nanoparticle-dispersed carbon catalysts and lactic acid yield (%) after hydrothermal conversion of

cellulose. The hydrothermal conversions were performed at 190 °C for 12 to 48 h.

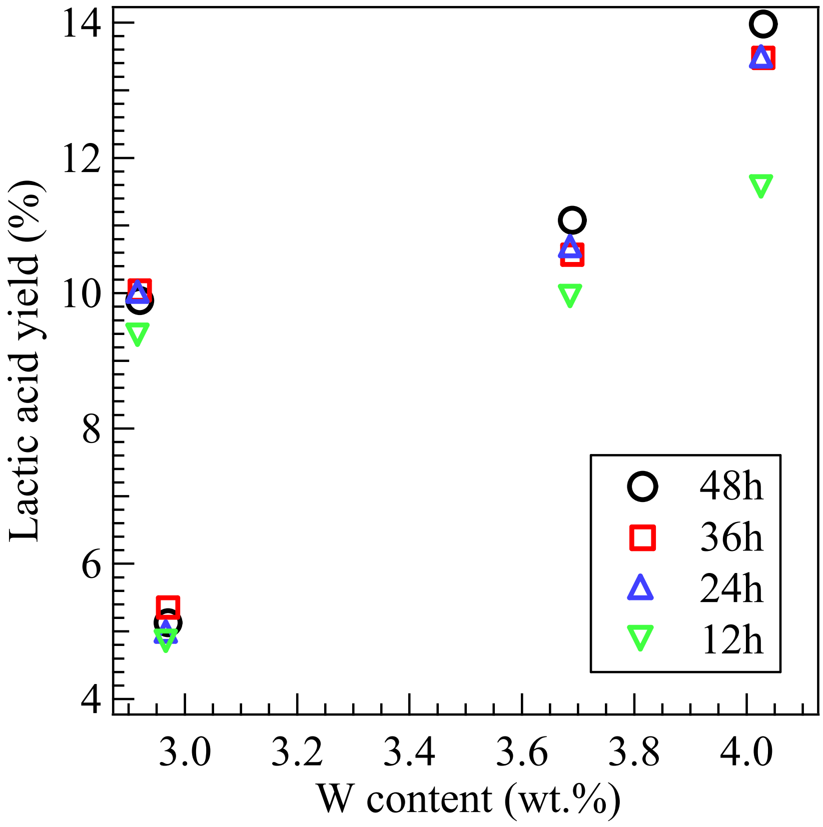

Figure S8: (a) SEM image and (b) EDS spectrum of the WC sample synthesized by SPP using a C electrode.

(a)

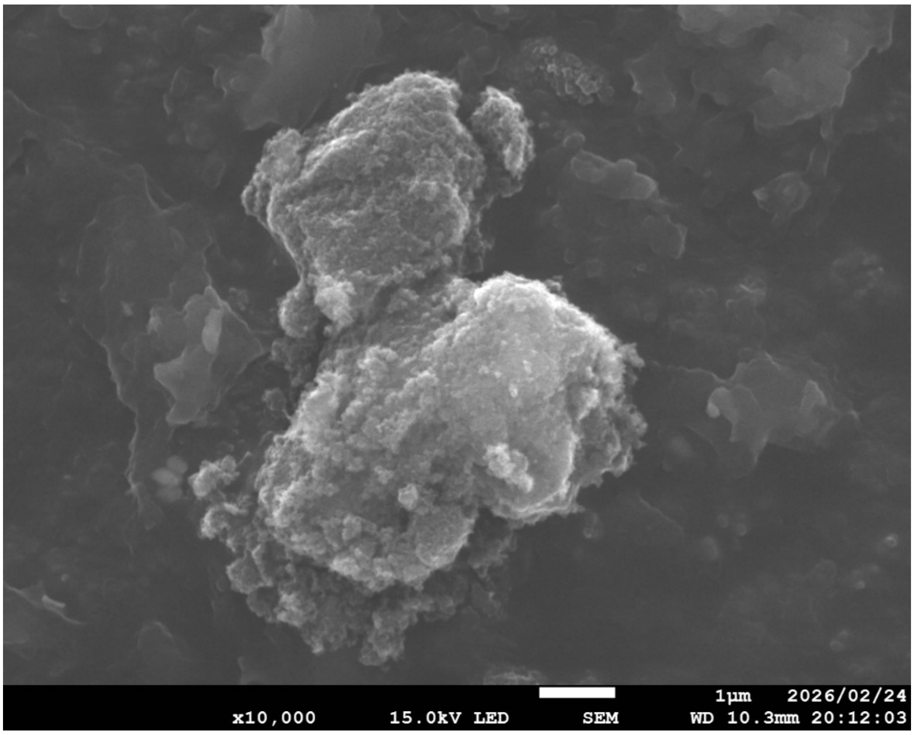

(b)

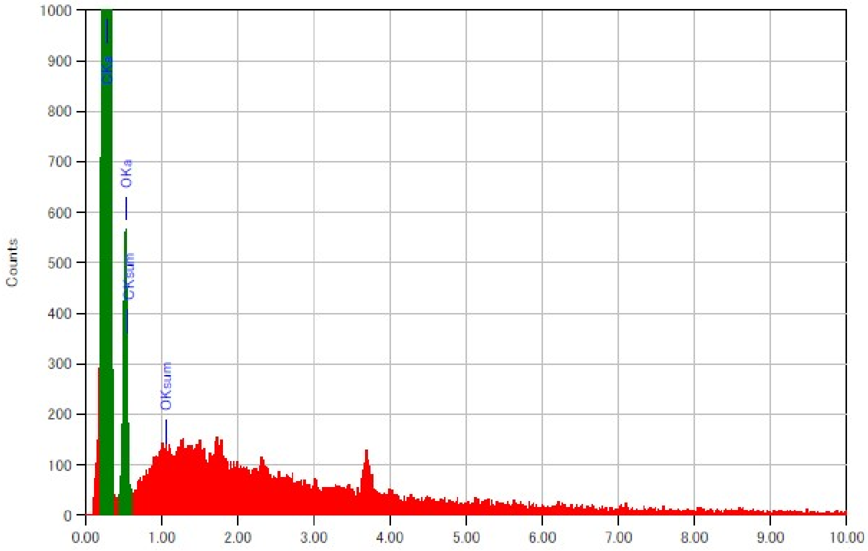

Figure S9. SEM images of the WC_{1-x}/C catalyst (a) before and (b-e) after catalytic cycles: (b) 1st, (c) 2nd, (d) 3rd, and (e) 4 th.

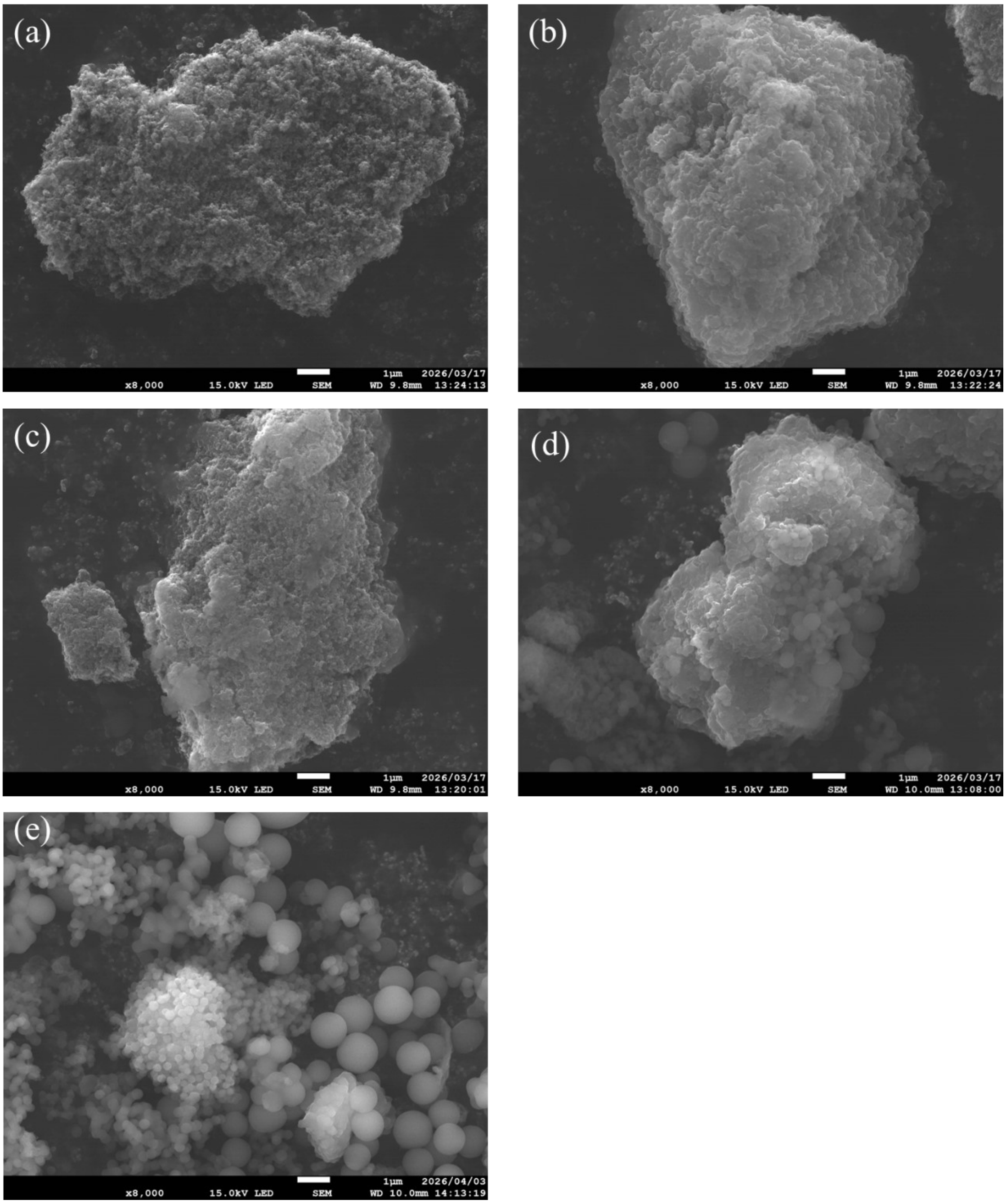

Figure S10. EDS analysis of the WC_{1-x}/C catalyst (a) before and (b-e) after catalytic cycles: (b) 1st, (c) 2nd, (d) 3rd, and (e) 4 th.

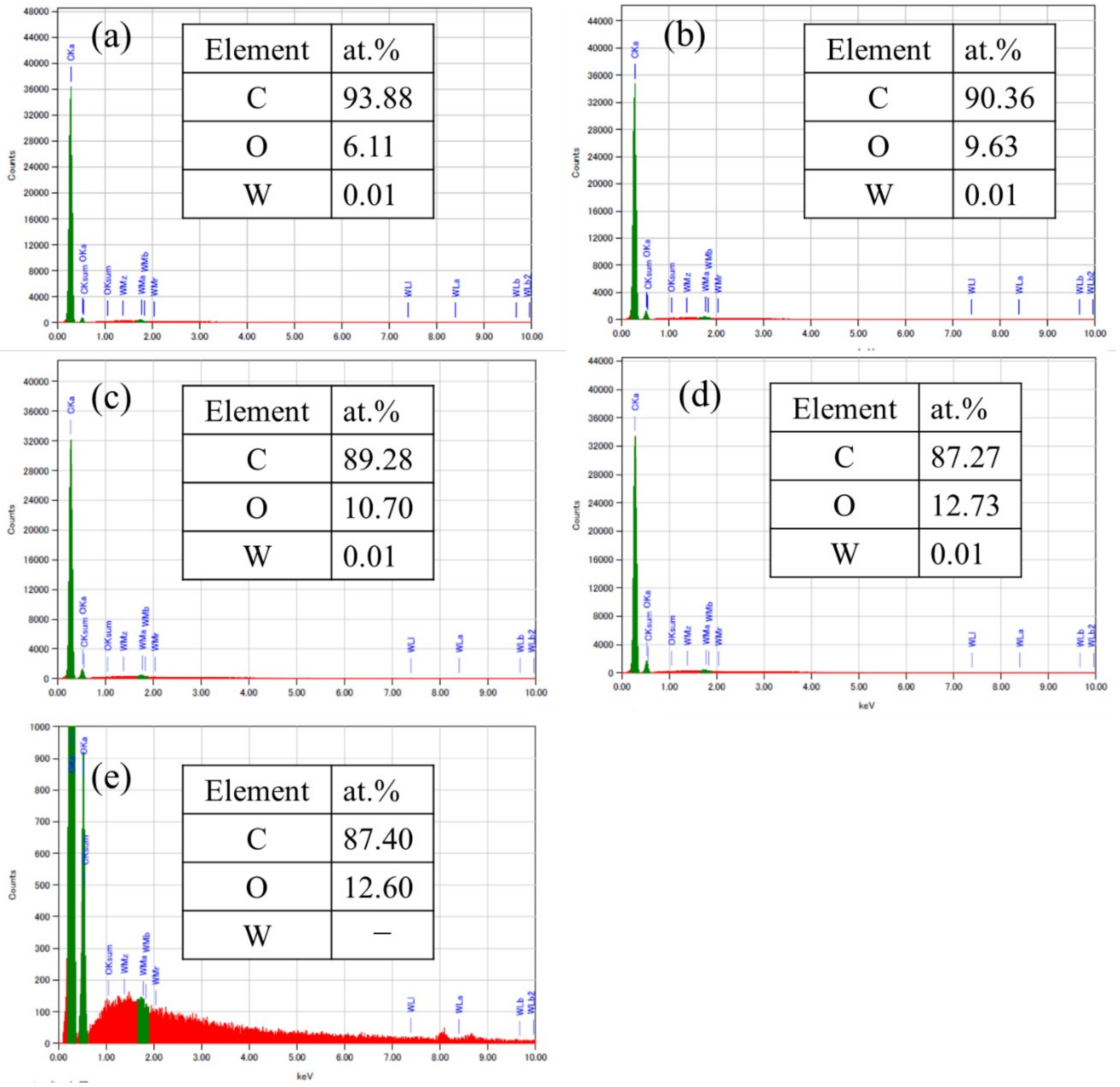

Fig. S11. XRD patterns of the WC_{1-x}/C catalyst before and after four catalytic cycles.

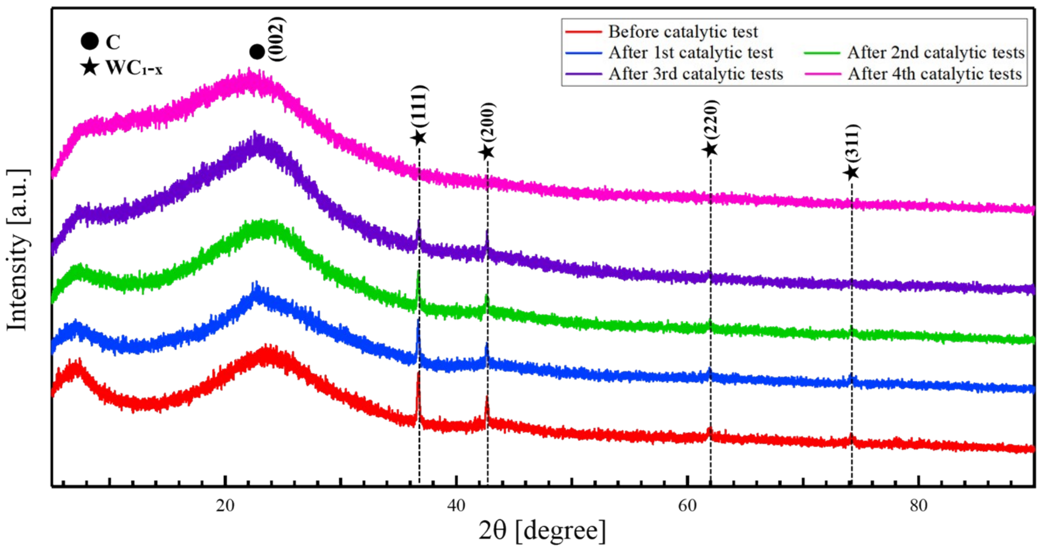

● C
★ WC1-x
●(002)
★(111)
★(200)
★(220)
★(311)
Before catalytic test
After 1st catalytic test
After 2nd catalytic tests
After 3rd catalytic tests
After 4th catalytic tests
Intensity [a.u.]
20
40
60
80
2θ [degree]
